# Impacts of temperature on recombination rate and meiotic success in thermotolerant and cold-tolerant yeast species

**DOI:** 10.1038/s41437-025-00778-6

**Published:** 2025-07-26

**Authors:** Jessica McNeill, Nathan Brandt, Enrique J. Schwarzkopf, Mili Jimenez, Caiti Smukowski Heil

**Affiliations:** Department of Biological Sciences, North Carolina State University, Raleigh, NC USA

**Keywords:** Evolutionary biology, Fungal genetics

## Abstract

Meiosis is required for the formation of gametes in all sexually reproducing species and the process is well conserved across the tree of life. However, meiosis is sensitive to a variety of external factors, which can impact chromosome pairing, recombination, and fertility. For example, the optimal temperature for successful meiosis varies between species of plants and animals. This suggests that meiosis is temperature sensitive, and that natural selection may act on variation in meiotic success as organisms adapt to different environmental conditions. To understand how temperature alters the successful completion of meiosis, we utilized two species of the budding yeast *Saccharomyces* with different temperature preferences: thermotolerant *Saccharomyces cerevisiae* and cold-tolerant *Saccharomyces uvarum*. We surveyed three metrics of meiosis: sporulation efficiency, spore viability, and recombination rate in multiple strains of each species. As per our predictions, the proportion of cells that complete meiosis and form spores is temperature sensitive, with thermotolerant *S. cerevisiae* having a higher temperature threshold for completion of meiosis than cold-tolerant *S. uvarum*. We confirmed previous observations that *S. cerevisiae* recombination rate varies between strains and across genomic regions, and add new results that *S. uvarum* has comparably high recombination rates. We find significant recombination rate plasticity due to temperature in *S. cerevisiae* and *S. uvarum*, in agreement with studies in animals and plants. Overall, these results suggest that meiotic thermal sensitivity is associated with organismal thermal tolerance and may even result in temporal reproductive isolation as populations diverge in thermal profiles.

## Introduction

The cell division process of meiosis is essential for all sexually reproducing species. While the core structures and processes are conserved across animals, plants, and fungi, genetic and environmental factors are known to mediate various aspects of meiosis, including chromosome pairing and recombination (Lenormand et al. 2016; Wilkins and Holliday, 2009). Temperature is the most thoroughly studied of these environmental variables, with optimal temperature for meiosis varying across plant and animal species, and evidence of meiotic failure at high and low temperature extremes (Bomblies et al. 2015).

Variation in optimal meiotic temperature may partly reflect environmental sensitivity of meiotic structures. For example, the synaptonemal complex, which forms between homologous chromosomes during meiotic prophase I and facilitates chromosome pairing and recombination, shows defects and instability at high temperatures (Bayliss and Riley 1972; Bilgir et al. 2013; Higgins et al. 2012; Loidl 1989; Nebel and Hackett 1961; Pao and Li 1948; Zheng et al. 2014). Defects of the synaptonemal complex at high temperatures are likely linked to chromosome pairing failure, which results in improper segregation of chromosomes and reduced fertility (Elliott 1955; Higgins et al. 2012; Loidl 1989; Pao and Li 1948; Yazawa et al. 2003). Genes encoding components of the synaptonemal complex and other meiotic proteins thus may be under selection as organisms experience different environmental conditions (Bomblies et al. 2015; Henderson and Bomblies 2021; Morgan et al. 2017). Indeed, selection on meiotic genes has been identified in *Drosophila*, mammals, and *Arabidopsis*, though explicit links with temperature or the environment are not clear (Dapper and Payseur 2019; Dumont 2020; Samuk et al. 2020; Turner et al. 2008; Wright et al. 2015). Long-standing laboratory studies in plants, animals, and yeast have documented recombination rate plasticity at different temperatures and support the connection between thermal sensitivity of meiotic processes and potential for meiotic evolution (Lim et al. 2008; Modliszewski et al. 2018; Morgan et al. 2017; Plough 1917; Stevison et al. 2017; Winbush and Singh 2021; Zhang et al. 2017). More recent studies in natural populations of *Drosophila* and *Arabidopsis* provide further correlational evidence, in which populations isolated from locations or seasons with different temperatures show differences in recombination rate (Dumont 2020; Samuk et al. 2020; Weitz et al. 2021).

One way to further elucidate the link between temperature and the evolution of meiosis is to investigate how temperature alters meiotic phenotypes within and between related species with different thermal niches. The budding yeast *Saccharomyces* is an ideal system to investigate this question, as optimal growth temperature is a defining characteristic delineating species of the genus. Temperature appears to be an important factor in defining *Saccharomyces* species ranges and ecology (Langdon et al. 2020; Leducq et al. 2014; Robinson et al. 2016), and while little is known to impact prezygotic isolation between species, there is evidence that divergent thermal profiles may maintain distinct lineages in sympatry (Gonçalves et al. 2011; Sampaio and Gonçalves 2008; Sweeney et al. 2004). The model system *Saccharomyces cerevisiae* is the most thermotolerant species, an apparent derived trait (Gonçalves et al. 2011; Molinet and Stelkens 2025; Peris et al. 2022; Salvadó et al. 2011). *S. cerevisiae* can grow in a wide range of temperatures, with a maximum temperature of 45.4 °C (Salvadó et al. 2011). While known for its role in human associated fermentations like wine, beer, bread, and sake, it can also be isolated from natural environments, including fruit, soil, and tree bark (Duan et al. 2018; Lee et al. 2022; Liti et al. 2009; Peris et al. 2022; Peter et al. 2018). Other species in the clade, like *Saccharomyces uvarum*, are more cold tolerant (Molinet and Stelkens 2025; Peris et al. 2022; Salvadó et al. 2011). *S. uvarum* has been isolated from cold-fermented beverages like wine and cider, and also is associated with tree bark (Almeida et al. 2014; Peris et al. 2022). Strains of *S. uvarum* have a maximum mitotic growth temperature of 36–38 °C (Salvadó et al. 2011; Sampaio and Gonçalves 2008), but some natural strain isolates appear more thermosensitive (Almeida et al. 2014).

Meiosis in *Saccharomyces* is facultative and is induced when nitrogen and fermentable carbon sources are depleted, but non-fermentative carbon sources remain (Jambhekar and Amon 2008; Zhao et al. 2018). Similar to gametogenesis in animals, the termination of the sexual cycle results in the formation of four haploid spores (a process called sporulation). The ability and speed of sporulation are variable and heritable, and alleles at several genes have been identified to contribute to variation in this trait (De Chiara et al. 2022; Gerke et al. 2006; Tomar et al. 2013). Once sporulated, the spore wall is resistant to a variety of stressors, and spores can remain in this dormant state for long periods of time. Sensing of glucose initiates the process of germination, and spores can resume asexual growth as haploids or mate to form diploids. Spore viability is intricately tied to successful chromosome pairing and recombination, with inviable spores often resulting from chromosome missegregation (Rogers et al. 2018).

While studies have identified genes and pathways implicated in mitotic temperature tolerance in *Saccharomyces* (AlZaben et al., (2021); Baker et al. 2019; Li et al. 2019; Weiss et al. 2018), meiotic thermal sensitivity is less understood. Common *S. cerevisiae* lab strains have variable sporulation at temperatures ranging from 15 °C to 34.5 °C, with strain backgrounds S288C and W303 having reduced sporulation at higher and lower temperatures relative to 30 °C (Elrod et al. 2009). A number of temperature-sensitive alleles that cause meiotic arrest have been identified, but this has mostly focused on mutagenesis of lab strains and not identifying naturally segregating alleles (Byers and Goetsch 1982; Davidow and Byers 1984; Esposito and Esposito 1969). As has been appreciated in a number of other organisms, temperature also changes the number and location of crossovers and non-crossover gene conversions in *S. cerevisiae* during meiosis (Cotton et al. 2009; Fan et al. 1995; Johnston and Mortimer 1967; Zhang et al. 2017). Together, these studies suggest that meiosis is thermosensitive in *S. cerevisiae*, and that leveraging the strain and species diversity of the *Saccharomyces* clade may help untangle how selection acts on meiosis to shape adaptation to new temperatures, and how meiotic temperature tolerance contributes to reproductive isolation.

Given the important role temperature plays in delineating *Saccharomyces* species ranges and periods of activity, we hypothesize that there may be differences in optimal temperature for meiosis between species with different thermal preferences. Here, we utilized strains of thermotolerant *S. cerevisiae* and cold-tolerant *S. uvarum* collected from a variety of ecological niches across the world (Table S1). We measured several meiotic phenotypes at a range of temperatures: the proportion of diploid cells that complete meiosis, the proportion of spores that are viable following meiosis, and recombination rate across multiple genomic intervals. We document variation within and between species in the successful completion of meiosis, and find that temperature affects recombination rate in both species.

## Results

### Mitotic growth recapitulates known thermal preferences in *S. cerevisiae* and *S. uvarum*

We selected 6 strains each of *S. cerevisiae* and *S. uvarum* that were isolated from different ecological niches and geographic locations (Table S1) (Almeida et al. 2014; Liti et al. 2009). We then created two sets of strains for further analysis. The first set of strains represents “pure” diploid strain backgrounds for each species. For the second set of strains, we crossed all *S. uvarum* and *S. cerevisiae* strains to fluorescently marked recombination tester strains with lab-strain backgrounds of CBS7001 and SK1, respectively (refer to “Methods”, Table S1). These tester strain crosses are thus heterozygous for a strain of interest and the tester strain, and are necessary for downstream measurement of recombination rate. To investigate the effect of temperature on the mitotic growth kinetics of these strains, we measured growth rate at three different temperatures (25 °C, 30 °C, 37 °C) (Fig. S1, Tables S4, 5). Microbial growth rate is both genetically and environmentally labile, varying widely between strains and conditions. Generally, we recapitulate known thermal preferences for both species. *S. uvarum* exhibited negligible growth at 37 °C for any strain except CBS7001, which had an average growth rate of 0.233 (SD 0.0755); thus, all *S. uvarum* growth rate measures at 37 °C were excluded from further analysis. *S. cerevisiae* strain YIIC17_E5/YIIC17_E5 also exhibited negligible growth at 37 °C, and was similarly omitted. In *S. uvarum*, temperature and heterozygosity from the tester-strain have significant effects on growth rate (ANOVA *p* < 0.0001 for temperature, heterozygosity), and strains grow faster at 30 °C than 25 °C degrees (Tukey’s HSD *p* < 0.05). “Pure” strains of *S. uvarum* generally exhibit higher growth rates at 25 °C compared to their tester-crosses (Tukey’s HSD *p* < 0.05). In *S. cerevisiae*, there is again a significant effect of temperature and heterozygosity on growth rate (ANOVA, *p* < 0.0001 for temperature, *p* = 0.0372 for heterozygosity). *S. cerevisiae* growth rates are lower at 25 °C compared to both 30 °C and 37 °C (Tukey’s HSD *p* < 0.05), but no significant difference in growth rate is identified between 30 °C and 37 °C. There is no difference between the growth rate of *S. cerevisiae* tester-crossed and “pure” strains at a given temperature. As predicted by known species thermal preferences, *S. cerevisiae* grows faster than *S. uvarum* at 25 °C and 30 °C (ANOVA, *p* < 0.0001 for temperature, species; Tukey’s HSD *p* < 0.05).

### High temperatures result in failure to complete meiosis in cold-tolerant *S. uvarum*

**Fig. 1:**
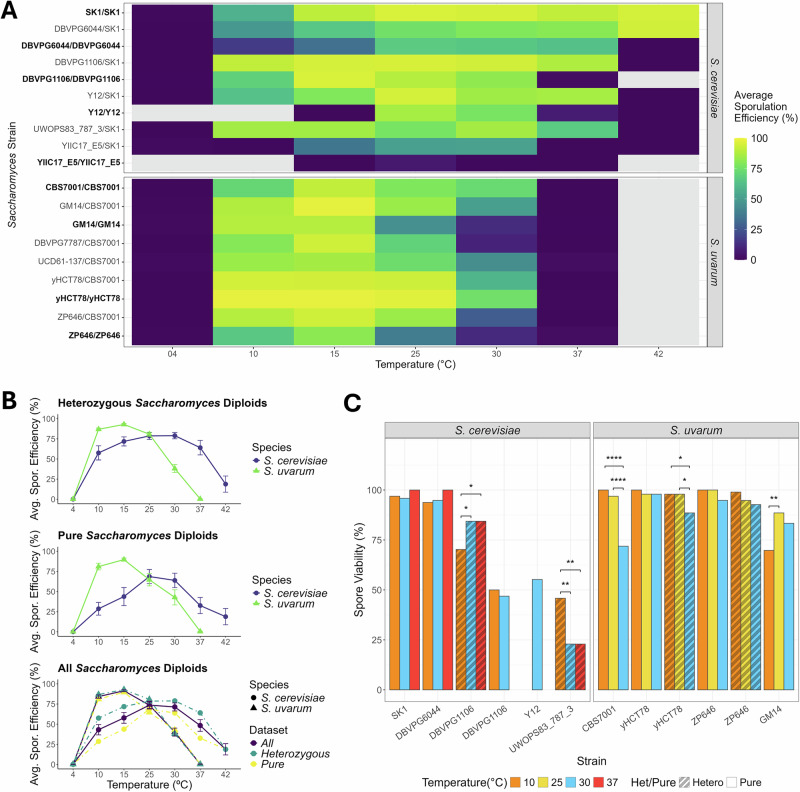
Sporulation efficiency and spore viability of all *S*. *cerevisiae* and *S. uvarum* strains. “Pure” strain diploids are bolded and distinguishable from their heterozygous counterparts by their listed genotype. **A** Heatmap displaying average sporulation efficiency (%) of each strain calculated from 3 biological replicates sporulated at each temperature for a minimum of 10 days. No data was collected for “pure” strains at temperatures above/below those where no appreciable spores were observed. Additionally, no data were collected for *S. uvarum* strains at 42 °C. **B** Average sporulation efficiency (%) of each species at each measured temperature. Plots display data of heterozygous strain, “pure” strain, and all species datasets, respectively. Error bars indicate standard error across all species’ strain replicates. For *S. cerevisiae* “pure” strains, an average sporulation efficiency of zero was assumed for temperatures measured not collected for some strains (4 °C, 10 °C, and 42 °C) due to failure at more moderate ends of the range. **C** Spore viability (%) calculated from at least 21 meioses (84 spores) of 6 selected strains from each species. Temperatures were selected to correspond with the known thermal preference and observed lower and upper boundaries of successful sporulation within each species. Stars denote significance as revealed through a Fisher's exact test (assuming a 95% confidence interval), followed with a post-hoc pairwise Fisher’s exact test (p-values corrected using Benjamini-Hochberg FDR method at a 5% cut-off).

To identify how temperature affects meiosis in *S. cerevisiae* and *S. uvarum*, we first measured the proportion of diploid cells that complete meiosis and form spores (sporulation efficiency) (Fig. 1A, Tables S6, S7). We induced meiosis in strains of each species at temperatures 4°, 10°, 15°, 25°, 30°, 37°, and 42 °C (*S. cerevisiae* strains only) (see “Methods”). There is extensive *S. cerevisiae* strain variation in ability to sporulate, and the length of time needed to sporulate, with several major loci known to contribute to variation (De Chiara et al. 2022; Gerke et al. 2006; Tomar et al. 2013). In accordance with previous research published by Raffoux et al. (2018b), we deemed an incubation period of at least 10 days sufficient to capture the maximum sporulation efficiency of all *S. cerevisiae* and *S. uvarum* strains at each temperature.

Overall, when we take an average sporulation efficiency of all strains from each species at each temperature, we see a parabolic effect of temperature on sporulation efficiency (Fig. 1B). The shape of the parabola is species dependent, with *S. cerevisiae* showing a peak in sporulation efficiency between 25 °C and 30 °C, and *S. uvarum* at 15 °C. No strains from either species produced appreciable (>3%) spores at 4 °C, establishing a lower thermal limit. We then constructed a logistic regression model to understand the effects of temperature on sporulation efficiency (Table 1). We corrected for heterozygosity due to tester background and for species, for which there is a significant positive effect of *S. uvarum* on sporulation efficiency. We identify a significant positive linear and negative quadratic effect of temperature on sporulation efficiency, with the quadratic term describing the parabolic shape of sporulation efficiency based on temperature. We also find that temperature affects strains differently, which we address below.

**Table 1:** Coefficients of a logistic regression model of sporulation efficiency.

	Estimate	Std. error	Z value	Pr(>|z|)
(Intercept)	−7.6058	1.0315	−7.3734	0.0000
Temperature	0.6882	0.0842	8.1750	0.0000
Temperature^2^	−0.0182	0.0021	−8.8585	0.0000
Species (*S. uvarum*)	3.6304	0.7785	4.6634	0.0000
Pure-strain diploid	−0.2157	0.7152	−0.3015	0.7630
Temperature:SK1	0.2896	0.0571	5.0682	0.0000
Temperature:DBVPG1106/SK1	0.2222	0.0516	4.3051	0.0000
Temperature:DBVPG1106	0.1644	0.0579	2.8368	0.0046
Temperature:DBVPG6044/SK1	0.2522	0.0527	4.7842	0.0000
Temperature:DBVPG6044	0.1594	0.0577	2.7619	0.0057
Temperature:Y12/SK1	0.2080	0.0511	4.0720	0.0000
Temperature:Y12	0.1258	0.0575	2.1865	0.0288
Temperature:YILC17_E5/SK1	0.0846	0.0507	1.6687	0.0952
Temperature:YILC17_E5	−0.0728	0.1200	−0.6070	0.5438
Temperature:UWOPS83_787_3/SK1	0.1943	0.0506	3.8393	0.0001
Temperature:GM14/CBS7001	−0.0062	0.0420	−0.1480	0.8824
Temperature:GM14	−0.0557	0.0532	−1.0476	0.2948
Temperature:yHCT78/CBS7001	0.0065	0.0424	0.1524	0.8789
Temperature:yHCT78	0.0342	0.0512	0.6679	0.5042
Temperature:ZP646/CBS7001	−0.0265	0.0418	−0.6342	0.5259
Temperature:ZP646	−0.0708	0.0546	−1.2959	0.1950
Temperature:UCD61-137/CBS7001	−0.0222	0.0418	−0.5326	0.5943
Temperature:DBVPG7787/CBS7001	−0.0518	0.0426	−1.2166	0.2237

We included whether a strain was heterozygous for a tester strain or a “pure” strain background as a correction in our model (indicated as Pure-strain diploid). Strains SK1, DBVPG1106, DBVPG6044, Y12, and YILC17_E5, and UWOPS83_787_3 are *S. cerevisiae* strains, either listed as a “pure” strain or crossed to tester SK1. Strains GM14, yHCT78, ZP646, UCD61-137, and DBVPG7787 are *S. uvarum* strains, either listed as a “pure” strain or crossed to tester CBS7001. Temperature:strain terms are in relation to strain CBS7001/CBS7001.

When compared to the *S. uvarum* strain CBS7001 as the baseline, we find a significant positive interaction of temperature with most strains of *S. cerevisiae*, consistent with higher sporulation efficiency at higher temperatures (Table 1). Most SK1 tester-crossed *S. cerevisiae* strains successfully sporulated within the range of 10–37 °C, with the exception of YIIC17_E5/SK1, which exhibited a far narrower range of only 15–30 °C (Fig. 1A). DBVPG6044/DBVPG6044, DBVPG1106/DBVPG1106, Y12/Y12, and YIIC17_E5/YIIC17_E5 exhibited lower efficiencies and smaller thermal ranges than their tester-crossed counterparts, with half failing to sporulate at 15 °C and most failing at 37 °C. SK1/SK1 exhibited the highest average sporulation efficiency of the species across all temperatures under survey (74.79% when averaged across all replicates, including 4 °C; all replicates were included in this calculation to evaluate the comparative robusticity of strain sporulation across all temperatures), and remained among *S. cerevisiae* strains with the highest average sporulation efficiency at each temperature. A high degree of consistent meiotic success is expected in SK1/SK1, as SK1 is a well-established lab strain often selected for meiotic study; such selection likely also contributes to the higher sporulation efficiency observed in the tester-crosses comparative to their “pure” counterparts.

While not significant in comparison to *S. uvarum* strain CBS7001, we find a negative interaction between temperature and *S. uvarum* strains on sporulation efficiency, consistent with higher sporulation efficiency at lower temperatures in this species (Table 1). CBS7001/CBS7001 reached a maximum average sporulation efficiency of 90.82% (SD = 2.91) at 15 °C, comparable to the maxima of most other *S. uvarum* strains. All *S. uvarum* CBS7001 tester-crossed strains produced spores within the range of 10–30 °C; however, average sporulation efficiency at 30 °C dropped as low as 9.54% (DBVPG7787/CBS7001, SD = 1.73). Average “pure” *S. uvarum* sporulation efficiencies were comparable to tester-crosses at 10 °C, with the exception of ZP646/ZP646, which exhibited a moderately lower measure*;* however, only CBS7001/CBS7001 and yHCT78/yHCT78 proved capable of producing significant spores at 30 °C, with ZP646/ZP646 and GM14/GM14 both failing to produce an average greater than 12.5% (11.48%, SD = 1.45 and 12.31%, SD = 1.97, respectively). Unlike *S. cerevisiae*, no *S. uvarum* strains produced appreciable spores at 37 °C. *S. uvarum* strains (both tester-crossed and “pure” strains) are more similar to one another in their sporulation efficiency and temperature range than strains of *S. cerevisiae*, which may be due to the history of the *S. cerevisiae* strains being used as haploids in lab studies. Overall, the interaction between strains and temperature supports our hypothesis that thermotolerant *S. cerevisiae* can produce spores at higher temperatures than cold-tolerant *S. uvarum*.

Because solid versus liquid media are known to influence sporulation efficiency, we also measured the sporulation efficiency of the tester strain backgrounds of both species (SK1/SK1 and CBS7001/CBS7001 for *S. cerevisiae* and *S. uvarum*, respectively) when cultured in liquid sporulation media. Average sporulation efficiencies for liquid SK1/SK1 cultures were higher than solid media at 10 °C and comparable at 15 °C; however, these measures appeared lower in liquid at higher temperatures, starting at 25 °C (Table S8). A decline in sporulation efficiency appears most notable at 37 °C, where liquid cultures averaged a sporulation efficiency of only 5.77% (SD = 2.79), contrasting the average measure of 90.88% (SD = 0.20) on solid media. CBS7001/CBS7001 had comparable average sporulation efficiency to solid cultures at both 15° and 25 °C. At 10 °C, sporulation efficiency measures were both higher and less varied, with an average of 90.32% (SD = 0.76) in liquid compared to 71.42% (SD = 10.81) on solid media. However, like SK1/SK1, CBS7001/CBS7001 sporulation measures also starkly declined in liquid media at the upper limit of the species’ thermal range, measuring an average sporulation efficiency at 30 °C of only 5.77% (SD = 2.64) compared to 78.36% (SD = 8.72) on solid media. While sporulation efficiency does differ depending on media type, we find the pattern of an increased thermal limit for sporulation in *S. cerevisiae* compared to *S. uvarum* holds true.

### The effect of temperature on spore viability is strain dependent in both species

Following measures of sporulation efficiency, we dissected tetrads from a subset of *S. cerevisiae* and *S. uvarum* strains to calculate the proportion of cells that survive meiosis and germinate (spore viability) (Fig. 1C, Tables S9–S12). Reduced spore viability is often the result of chromosome missegregation and aneuploidy (Rogers et al. 2018), which has also been linked to temperature sensitivity, though not yet in yeast (Henderson and Bomblies 2021). We induced meiosis at 10°, 30°, and 37 °C for *S. cerevisiae* strains, and 10°, 25°, 30 °C for *S. uvarum -* temperature ranges that correspond with each species’ known thermal preference, flanked by temperatures approaching the observed lower and upper boundaries of successful sporulation within each species. *S. uvarum* strains CBS7001/CBS7001 and yHCT78/CBS7001 show evidence of reduced spore viability with increasing temperature; however, this trend does not persist in other strains, with no significant difference detected across temperatures for ZP646/CBS7001, yHCT78/yHCT78, or ZP646/ZP646, and reduced viability observed in GM14/GM14 at 10 °C comparative to 25 °C (Table S12). This suggests a strain-dependent response of spore viability to temperature in *S. uvarum*.

Similar strain variance is observed in *S. cerevisiae* spore viability. While temperature did not have a significant effect on spore viability in SK1/SK1, lower temperature influences viability in DBVPG1106/SK1 and UWOPS83_787_3/SK1 strains (Table S10). The nature of this impact is strain-dependent, with DBVPG1106/SK1 exhibiting decreased spore viability at colder temperatures, and UWOPS83_787_3/SK1, increased. The cold preference observed in the UWOPS83_787_3/SK1 cross is especially interesting, as UWOPS83_787_3 has a documented geographical source location of the Bahamas. Due to the narrower thermal range of sporulation observed in all *S.cerevisiae* “pure” strains, collecting data at all temperatures for DBVPG6044/DBVPG6044, DBVPG1106/DBVPG1106, and Y12/Y12 was not possible. While Y/12Y12 exhibited a spore viability of only 55.21% at its singular 30 °C measure, DBVPG6044/DBVPG6044 exhibited high viability with no significant difference at all three temperatures. DBVPG1106/DBVPG1106 did not sporulate at 37 °C, and exhibited no significant change in spore viability from 10° to 30 °C; although, both measures are notably lower than those of DBVPG1106/SK1, suggesting that the SK1 strain may be masking additional strain variation in the SK1-crossed diploids.

### Temperature affects recombination rate in *S. cerevisiae* and *S. uvarum*

**Fig. 2:**
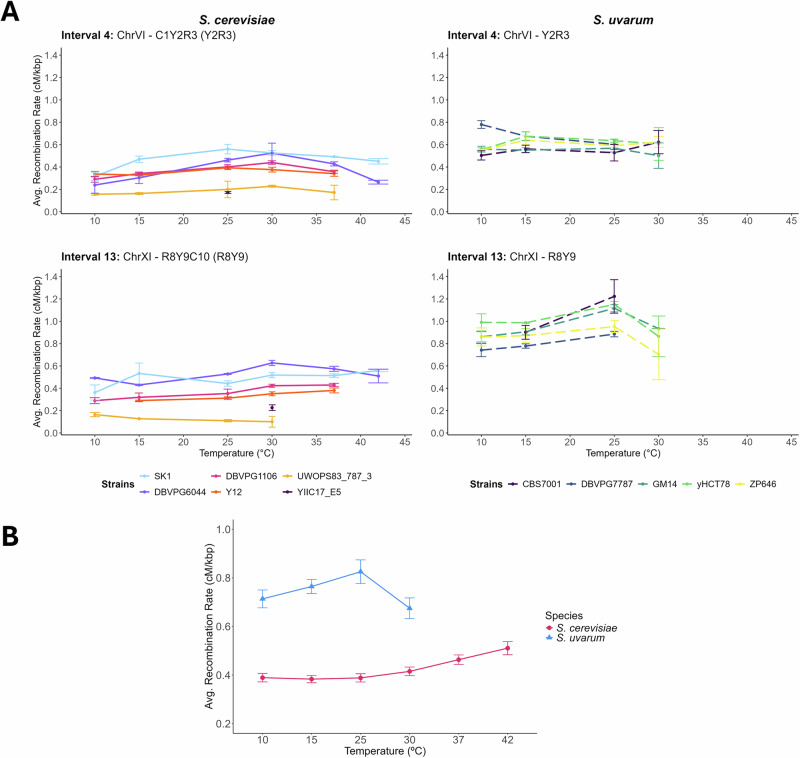
Average recombination rate (cM/kb) calculated for the syntenic intervals of 4 (on ChrVI) and 13 (on ChrXI) in *S. cerevisiae* and *S.uvarum* across all measured temperatures. *S. cerevisiae* recombination rate estimates were corrected for fluorescence extinction using a maximum likelihood model derived in Raffoux et al. (2018a). *S. uvarum* recombination rate estimates were corrected for fluorescence extinction using a derivative of this script adjusted for only two fluorescent loci. No data was collected for *S. uvarum* strains at 42 °C. In strain-level comparisons (**A**), strain name refers to the parent strain crossed with each fluorescent tester to produce a diploid for sporulation. Error bars indicate standard deviation above and below the mean, as calculated between biological replicates of each strain. In species-level comparisons, (**B**) error bars indicate standard error across all species’ strain replicates.

Finally, we measured the effect of temperature on recombination rate. We utilized the set of tester-crosses, with *S. cerevisiae* SK1 strains marked with fluorescent markers (Raffoux et al. 2018a), and fluorescent *S. uvarum* CBS7001 strains created to correspond to syntenic intervals in *S. cerevisiae* for comparison. We induced meiosis for all crosses at temperatures 10°, 15°, 25°, 30°, and 37 °C, and 42 °C (*S. cerevisiae* only) for 10 days, as above. After 10 days, sporulated cultures were enriched for spores and analyzed for recombination using flow cytometry. As this methodology directly observes meiotic products and is not reliant on spore growth, this analysis includes data from both viable and non-viable spores (Raffoux et al. 2018a). Altogether, we measured recombination rate for six strains (crossed to SK1 tester) of *S. cerevisiae* in 10 genomic intervals (chromosomes I, VI, and XI) at six temperatures, and five strains (crossed to CBS7001 tester) of *S. uvarum* in two genomic intervals (chromosomes VI and XI) at four temperatures (Figs. 2, S2–S6, Tables S2, 3, S13, 14). We excluded several strain/interval/temperature combinations due to low sporulation or other technical issues.

We then constructed a Gaussian generalized linear model to understand recombination rate as a function of temperature (Table 2). We find a positive linear effect and negative quadratic effect of temperature on recombination rate, with the quadratic term capturing non-linear variation in recombination rate due to temperature. This is consistent with studies in many plants and animals that temperature influences recombination rate. We also find a significant effect of species, with *S. cerevisiae* recombination rates significantly lower than *S. uvarum* (Fig. 2, Table 2, Tables S13, 14). *S. uvarum* recombination rates peaks at 25 °C, with lower recombination rates at higher and lower temperatures, whereas *S. cerevisiae* recombination rates increase with temperature, peaking at 42 °C (Fig. 2B). Interestingly, the temperatures with the highest sporulation efficiency and the temperatures with the highest recombination rates differ for both species, with optimal sporulation efficiency occurring at lower temperatures than highest recombination rates (Figs. 1B, 2B).

**Table 2:** Regression coefficients of a linear model of recombination rate as a function of temperature.

	Estimate	Std. error	*t* value	Pr(>|*t*|)
(Intercept)	0.7295	0.0420	17.3503	0.0000
Species (*S. cerevisiae*)	−0.4267	0.0411	−10.3708	0.0000
Temperature	0.0111	0.0031	3.6325	0.0003
Temperature^2^	−0.0002	0.0001	−4.4181	0.0000
Interval Y2R3	−0.1467	0.0165	−8.8724	0.0000
Interval C1Y2	0.0760	0.0199	3.8239	0.0001
Interval C3Y4	0.1228	0.0201	6.0993	0.0000
Interval C7R8	−0.2272	0.0198	−11.4515	0.0000
Interval R2C3	0.0582	0.0201	2.8890	0.0040
Interval R3Y4	−0.1987	0.0196	−10.1352	0.0000
Interval Y4C5	0.1812	0.0196	9.2444	0.0000
Interval Y6C7	−0.0725	0.0198	−3.6566	0.0003
Interval Y9C10	−0.0216	0.0204	−1.0587	0.2900
Temperature:SK1	0.0061	0.0023	2.6131	0.0091
Temperature:DBVPG1106	0.0015	0.0023	0.6472	0.5177
Temperature:DBVPG6044	0.0041	0.0023	1.7308	0.0838
Temperature:Y12	0.0008	0.0023	0.3493	0.7269
Temperature:YIIC17_E5	−0.0074	0.0024	−3.0940	0.0020
Temperature:UWOPS83_787_3	−0.0074	0.0024	−3.1211	0.0019
Temperature:GM14	−0.0010	0.0020	−0.4959	0.6201
Temperature:yHCT78	0.0016	0.0020	0.7784	0.4365
Temperature:ZP646	−0.0022	0.0020	−1.0934	0.2745
Temperature:DBVPG7787	−0.0017	0.0024	−0.7133	0.4758

Intervals were included as a correction in this model, and more information about intervals can be found in Table S2. Strains SK1, DBVPG1106, DBVPG6044, Y12, and YILC17_E5, and UWOPS83_787_3 are *S. cerevisiae* strains, which are all crossed to tester SK1. Strains GM14, yHCT78, ZP646, and DBVPG7787 are *S. uvarum* strains, which are all crossed to tester CBS7001. Temperature:strain terms are in relation to strain CBS7001.

After correcting for species, temperature effects, and genomic region (interval), we find that most strains of both species respond similarly to temperature (Table 2). There are a few exceptions for *S. cerevisiae* strains: SK1 has a more pronounced reaction to temperature, whereas strains UWOPS83_787_3 and YIIC17_E5 have the opposite reaction to that of other *S. cerevisiae* strains. These strains have the highest and lowest recombination rates, respectively across temperatures (Figs. S6, S2–S5). *S. uvarum* strains had a similar response to temperature. Strain yHCT78, isolated from tree bark at a vineyard in Missouri, USA, has the highest recombination rates averaged across all temperatures (Interval 4: 0.62 cM/kb, Interval 13: 1.01 cM/kb), whereas strains with the lowest recombination rates depend on the interval. While previous work has identified that genetic background influences recombination rate, and we find here that temperature influences recombination rate, temperature-strain interactions are complex.

## Discussion

Sympatric species of *Saccharomyces* isolated from the same microhabitat have different thermal niches, as measured by their ability to grow mitotically under warm or cold temperatures (Gonçalves et al. 2011; Sampaio and Gonçalves 2008; Sweeney et al. 2004). While most *Saccharomyces* species can grow at a wide range of temperatures, their optimal growth temperature and maximum growth temperature are clearly delineated, with *S. cerevisiae* outcompeting other species at warm temperatures and species like *S. kudriavzevii* and *S. uvarum* outcompeting at cold temperatures (typically at or below 12 °C). Our study supports these observations, incorporating strains isolated from a variety of ecological niches, with *S. uvarum* exhibiting lower growth rates to *S. cerevisiae* at 25 °C and 30 °C, and unable to grow at 37 °C. We interpret these results to be consistent with previous observations that *S. cerevisiae* has increased thermotolerance in comparison to other *Saccharomyces* species (Molinet and Stelkens 2025; Peris et al. 2022; Salvadó et al. 2011).

In comparing sporulation and spore viability between thermotolerant and cold-tolerant species, we predicted that as *S. cerevisiae* evolved to be increasingly thermotolerant, meiotic structures and processes would have increased thermal tolerance. Indeed, we do see an ability for *S. cerevisiae* to sporulate at higher temperatures than *S. uvarum* (highest percentage of sporulation for *S. uvarum* 15 °C; *S. cerevisiae* 25–30 °C). In contrast, temperature does not seem to consistently affect spore viability in our measured strains, though our estimates of spore viability might be skewed as we only assessed spore viability for strains in which we could collect complete tetrads. Many strains had a large proportion of monads, dyads, and other abnormal spore morphologies, some of which worsened with departure from the maximum sporulation temperature.

While an ideal temperature is apparent for sporulation, what an ideal temperature is for recombination rate is less clear. The effect of temperature on recombination rate is variable between different organisms, with a number of species displaying a U-shaped curve with the lowest recombination rate at intermediate temperature conditions with elevated recombination rates at low and high temperatures (Bomblies et al. 2015; Henderson and Bomblies 2021). We do not find a U-shaped pattern of recombination rate; instead, we generally find an increase in recombination rate with temperature in both species (though *S. uvarum* recombination rate declines at the highest temperature able to be measured). Interestingly, the temperature that maximizes sporulation efficiency and the temperature that maximizes recombination rate are different for both species, perhaps suggesting that temperature is impacting different phases of meiosis in distinct ways. The dip in recombination rates seen at 30 °C in *S. uvarum* might indicate that meiotic processes are starting to fail under temperature stress, but no similar observation is seen at the highest temperatures in *S. cerevisiae*, so this is hard to interpret, and more mechanistic studies are needed to assess this. The flow cytometry method we utilized measures recombination in both viable and inviable spores, and it is possible that this could complicate our results if inviable spores had different patterns of recombination due to temperature. Again, this requires further investigation.

We observed an approximately 1.2–1.3 fold change in average recombination rates due to temperature (*S. cerevisiae*: 0.383–0.511 cM/kb; *S. uvarum* 0.675–0.825 cM/kb). Our results are similar in magnitude as the 1.2–1.4 fold change in *Arabidopsis* (Lloyd et al. 2018; Modliszewski et al. 2018) and lower than the 2–3 fold change in *Drosophila* and *C. elegans* (Plough 1917; Rose and Baillie 1979; Smith 1936; Stevison et al. 2017). Several relevant hypotheses have been proposed to explain recombination rate evolution and plasticity. For example, some studies have shown that short-term selection for traits including geotaxis, pesticide resistance, parasite resistance, and temperature has resulted in increased recombination rates (Aggarwal et al. 2019; Kohl and Singh 2018; Korol and Iliadi 1994). We might extend this to predict that selection on increased temperature tolerance in *S. cerevisiae* over time would lead to higher recombination rates in *S. cerevisiae* than *S. uvarum*, but we saw the opposite pattern. *S. uvarum* strains have higher recombination rates in the two syntenic intervals measured, which is in keeping with our recent work finding comparably high genome-wide recombination rates in *S. uvarum* (Schwarzkopf et al. 2024). However, it is not clear that short-term selection studies would apply to broader time scales (Bursell et al. 2024), and the comparison between recombination rate evolution in *S. cerevisiae* and *S. uvarum* is complicated by their 20 million years of divergence. Alternatively, under the hypothesis of fitness associated recombination rate plasticity, resistant genotypes may respond less than sensitive genotypes (Aggarwal et al. 2019; Rybnikov et al. 2017). For example, in a study that compared recombination rates of heat resistant and cold resistant tomato lines at different temperatures, the heat resistant line showed a more moderate increase in recombination rate than cold resistant lines at high temperature, and vice versa (Rybnikov et al. 2017). Our data are not consistent with this hypothesis either.

Proxy measures of meiotic success may also be impacted by other variables, like genetic background and additional environmental factors like sporulation on liquid vs. solid media and amount of sporulation time, which are known to affect sporulation efficiency and viability (Elrod et al. 2009). We do note that sporulation in liquid results in sporulation failure at a lower temperature than sporulation on solid media; however, the pattern of effect of temperature on sporulation between species remains. We also find certain strains to have particularly low sporulation efficiency and recombination rates, consistent with previous studies (De Chiara et al. 2022; Gallone et al. 2016; Raffoux et al. 2018b), which may be attributable to mutations in meiotic genes and/or structural variants. Previous work also documented an effect of sequence similarity between tester and strain of interest on recombination rate (Raffoux et al. 2018b), and it is possible that “pure” strains may have more pronounced temperature responses, but this is quite difficult to measure empirically.

Overall, our data further advance the idea that divergent thermal niches in *Saccharomyces* may be reinforced by meiotic failure at non-permissive temperatures, which provides a potential temporal reproductive isolating barrier between divergent populations and species. Prezygotic reproductive isolation barriers like temporal isolation between diverging populations may arise as a byproduct of local adaptation. For example, differences in flowering time as a result of selection due to varying soil moisture, altitude, and other climatic variables are a striking and highly repeated observation of temporal variation reducing gene flow (Lowry et al. 2008; MacNair and Gardner 1998; Vasek and Sauer 1971). While species in *Saccharomyces* have strong postzygotic isolation, prezygotic isolation is not well understood (Ono et al. 2020). Mating pheromones are conserved across all *Saccharomyces* (Rogers et al. 2015), and mate choice assays show little to no preference for conspecifics (Murphy et al. 2006; Naumov et al. 2000). Mating and germination timing do differ in sympatric species (Murphy and Zeyl 2012), and it has been hypothesized that developmental timing and periods of activity may differ over the course of day or year (Sampaio and Gonçalves 2008). Our results support this hypothesis, in which cold-tolerant species would be unable to produce spores at certain times of the year.

Because temperature has such a strong influence on a wide variety of cellular processes and organismal behavior and survival, it has been studied extensively in a wide array of species. Organismal thermal tolerance across a range of temperatures is commonly used to measure and estimate range boundaries and predict response to climate change (Wooliver et al. 2022). However, many of these studies focus on parameters of growth that are not related to the sexual cycle, and an increasing number of studies support that an organism’s “thermal fertility limit” (TFL) is lower than its “critical thermal limit” (CTL) (Iossa 2019; van Heerwaarden and Sgrò 2021; Walsh et al. 2019). Our results add to these data, supporting that parental temperature growth maximums may overestimate an organism’s thermal niche, as meiotic failure occurs at a temperature below that of which it can sustain growth. We thus support recent work calling for measuring organisms’ thermal fertility limit to better understand population persistence in the face of environmental change (Walsh et al. 2019).

Finally, while meiosis has long been recognized to be sensitive to temperature, thermal sensitivity is rarely measured within species or between closely related species. Our documented variation of meiotic thermal sensitivity within and between species sets up a powerful system to test how meiotic thermal sensitivity can evolve. As the climate changes, this is likely to be a key parameter in determining species resiliency and long-term survival.

## Materials and methods

### Strains

A list of yeast strains is included in Table S1. Selected *S. cerevisiae* strains are part of the SGRP collection (Liti et al. 2009). The *S. uvarum* strains were obtained from the Portuguese Yeast Culture Center and courtesy of Chris Hittinger (Almeida et al. 2014). *S. uvarum* strains had their *HO* locus replaced with a KanMX or HygMX marker using a modified version of the high-efficiency yeast transformation using the LiAc/SS carrier DNA/PEG method with a heat shock temperature of 37 °C for 45 minutes. The markers were amplified using the plasmids and primer sets detailed in Table S3. Single colonies were selected from the transformation plates, sporulated, and dissected on YPD plates using a Singer SporePlay+ microscope (Singer Instruments). The plates were incubated at room temperature for 2 days and then replica plated to test for proper segregation of antibiotic marker and mating type within individual tetrads.

“Pure” *S. uvarum* strain representatives were obtained from the Portuguese Yeast Culture Collection. In *S. cerevisiae*, “pure” strains include a homozygous DBVPG6044 diploid from the Peter et al. (2018) 1011 collection (Standardized name: AKI), a cross of a Y12 haploid strain from the 1011 collection (Standardized name: ACK) with another haploid of the same strain, and homozygous DBVPG1106 and YIIC17_E5 strains constructed from haploids utilized elsewhere in this paper through mating type switching and crossing. To construct homozygous DBVPG1106 diploids, MAT-a haploids were transformed with the pHRW40 plasmid (Table S1). Using a protocol adapted from Peris et al. (2020), a mating type switch was induced by culturing cells in doxycycline. Plasmid loss was confirmed after passaging in non-selective media. Homozygous YIIC17_E5 diploids were constructed similarly using the plasmid p46 (Table S1), with transformants cultured in SC+Galactose (Dunham et al. 2015). Same-strain haploids were then crossed via micromanipulation. The “pure” strain identity of resulting diploids was confirmed through Sanger sequencing and SNP comparisons of the chrI, locus 4 region (primers in Table S3). Attempts were made to create a homozygous UWOPS83_787_3 diploid, but were ultimately unsuccessful.

### Measuring recombination rate

*S. cerevisiae* strains for measuring recombination rate were obtained courtesy of Matthieu Falque (Raffoux et al. 2018a, 2018b). This set consists of an array of SK1 haploid strains (Table S1) built to contain three fluorophores spaced approximately 30 cM apart within regions of one of three chromosomes (chrI, chrVI, or chrXI, Table S2). Six *S. cerevisiae* strains that showed variation in recombination rate in previous work (Raffoux et al. 2018b) were selected from the SGRP collection (Table S1). Haploids of these six strains were crossed via micromanipulation to fluorescent strains, and diploids were confirmed by halo assay. Following Raffoux et al., diploids were cultured overnight in 5 mL of liquid YPD (1% yeast extract, 2% peptone, 2% dextrose) at 30 °C. The following morning, each culture was transferred to a 15 mL conical tube and centrifuged at 2000 rpm for 2 min. Cell pellets were resuspended in 450 μL ddH_2_O, and 150 μL of each mixture was spread onto solid SPM plates (1% Potassium Acetate, 0.1% yeast extract, 2% agar, 0.05% dextrose) in triplicate. Plates were incubated at 4, 10, 15, 25, 30, 37, and 42 °C for 10 days. All samples were prepared for spore enrichment within the subsequent 3 days. To eliminate vegetative cells and enrich spores for recombination analysis, a methodology established by Raffoux et al. was adapted with slight alteration. A quarter to a half of the lawn of each successfully-sporulated plate was harvested with a bent pipette tip and suspended in a 1.5 mL tube containing 750 μL of ddH_2_O with 5 mg/mL 20 T Zymolyase, after which 100 μL of glass beads were added. To disrupt tetrads, this tube was vortexed for 1 minute at a frequency of 3000 rpm using a Disruptor Genie, incubated for 60 min at 30 °C, then subjected to another 3000 rpm vortex for 1 min. The liquid portion of each mixture was then transferred into a new 1.5 mL tube and centrifuged for 5 min at 4500 rpm. The resulting pellet was resuspended in 200 μL of ddH_2_O. This suspension was then discarded, as it contained primarily vegetative cells. Spores left adhering to the tube plastic were stripped by adding a solution of 600 uL ddH_2_O with 0.01% NONIDET NP40 and vortexing for 30 seconds. Upon analysis on the flow cytometer, this concentrated spore suspension was diluted with up to 1 mL of 1x PBS if necessary. Each culture was run on a Thermo Fisher Attune flow cytometer (University of North Carolina, Chapel Hill Flow Cytometry Core). To capture a population of spores, data were gated based on forward scatter and side scatter, then subsequently gated for single cells. All data yielding less than 2000 events within this single cell gate were discarded prior to further analysis. To confirm that the population of spores being captured was devoid of vegetative cells, mixed populations of vegetative and fluorescent spores, as well as vegetative cells, were analyzed as per Raffoux et al. (2018a). Our spore enrichment protocol yielded around 1% carryover of vegetative cells. Distinct fluorescent populations were gated further to extract numeric counts of all possible genotypes within the total spore population; in accordance with the protocol derived in Raffoux et al. 2018a, these counts could be input into a maximum likelihood model to estimate recombination rate while controlling for fluorescence extinction (Raffoux et al. 2018a). If distinct fluorescent populations could not be resolved for a sample following single-cell gating, the data were discarded. An attempt was made to analyze all strain-by-temperature combinations, but insufficient spores and poorly resolved fluorescent populations were produced for certain strain backgrounds and temperatures (Tables S6, 7, S11, 12).

To measure recombination rate in *S. uvarum*, two strains with two fluorophores on regions of either chrVI or chrXI were constructed using CBS7001 as a strain background (Tables S1, S2). These intervals were designed to be homologous and syntenic to intervals in *S. cerevisiae*. Multiple attempts were made to create a third strain with syntenic intervals on chrI, but a fluorescent marker was unable to be integrated at locus 3. Strains were constructed using templates and primers detailed in Table S3. *S. uvarum* strains were transformed with appropriate mCherry or YFP marker cassette following the same protocol as was used for the *HO* locus. Correct integration was confirmed via PCR and flow cytometry. mCherry and YFP strains were crossed and sporulated, and haploids of each mating type were confirmed to have both markers via PCR and flow cytometry. Haploids were then mated via micromanipulation to 6 *S. uvarum* strains of interest, and checked for diploidy by halo assay. Diploids were cultured overnight in 5 mL of liquid YPD at 25 °C, plated in triplicate on solid SPM plates (as above), and incubated at 4, 10, 15, 25, 30, and 37 °C for 10 days. The same spore enrichment and flow cytometry protocol was followed as above, accommodating two fluorescent markers per strain rather than three (as in *S. cerevisiae)*. An analogous maximum likelihood method was derived from the original Raffoux et al. 2018a script to estimate recombination rate while controlling for fluorescence extinction at only two fluorescent loci (Text S2).

### Measuring growth rate

Strains were grown in YPD overnight at room temperature. The following day, cultures were diluted to have a starting optical density (OD) of 0.1 or less and inoculated in 96 well plates at a volume of 200 μL. Growth curves were calculated for each strain at 25 °C, 30 °C, and 37 °C using a BioTek Epoch 96-well plate reader measuring the OD at 600 nm. Strains were measured in 6 replicates (2 technical replicates for each of 3 biological replicates) in different wells of the plate for each run to account for plate positioning effects. OD readings were collected every 15 minutes over a 48-hour period with continuous dual orbital shaking. Growth was analyzed using the R package “growthcurver” to calculate growth rate (r) (Sprouffske and Wagner 2016).

### Sporulation efficiency

Strains were sporulated in triplicate at 4, 10, 15, 25, 30, 37, and 42 °C (*S. cerevisiae* only) following the same protocol detailed above. After a 10-day incubation period, all measures were taken within 3 days. A quarter of the lawn of each plate was harvested using a bent pipette tip and suspended in 1 mL ddH_2_O. 10 μL of this suspension was placed onto a hemocytometer and investigated under a light microscope. If cell concentration remained too dense to facilitate clear counting, additional dilutions were performed. For each sample, two counts were taken of the number of spores with visible asci (monads, dyads, and tetrads) within an observed population of at least 200 cells. Proportions from these counts were then averaged to control for variation in spore calls between the two counts. Sporulation efficiency was calculated from these averages as the percentage of spores with visible asci within the total population observed for each sample.

### Liquid sporulation

SK1/SK1 (*S. cerevisiae*) and CBS7001/CBS7001 (*S. uvarum*) were measured for comparative sporulation efficiency in liquid SPM media vs. solid media at 10, 15, 25, and 30 °C. SK1/SK1 was also measured at 37 °C. Strains were sporulated using a protocol derived from (Trainor et al. 2021). *S. uvarum* and *S. cerevisiae* strains were first streaked out onto YPD plates (1% yeast extract, 2% peptone, 2% dextrose, 2% agar) and grown for ~48–72 h at 25 °C and 30 °C, respectively. A single colony was selected from each plate, moved to 5 mL liquid YPA (1% yeast extract, 2% peptone, 2% potassium acetate), and placed in a spinner to grow overnight at these same respective temperatures. This process was performed in triplicate to include 3 biological replicates for each cross at each temperature. The following day, a measure of OD600 was taken for each culture. Approximately 6 × 10^7^ cells were removed from each culture, spun down (~2500 rpm for 1 min), and washed with sterile ddH_2_O. Cells were then resuspended in 1 mL liquid SPM, and left to sporulate with agitation at a designated temperature for 10 days. After this 10-day incubation, 100 µl of each sporulated sample was diluted in 1 mL of sterile water, vortexed, and sonicated thoroughly. Cells were counted, and percent sporulated cells calculated in the same method as described above.

### Spore viability

Strains were sporulated for 10 days at temperatures 10 °C, 25 °C, and 30 °C (*S. uvarum*) or 10 °C, 30 °C, and 37 °C (*S. cerevisiae*). A patch of cells from the lawn of the solid SPM plate was harvested with a bent pipette tip, suspended in 50 μL of ddH_2_O, and centrifuged at 2000 rpm for 1 minute. The pellet was resuspended in a mixture of 25 μL ddH_2_O and 2 μL of 5 mg/mL 20 T Zymolyase (the equivalent of 1 U). This suspension was incubated in a 37 °C water bath for 10 min, then 500 μL of ddH_2_O was added to halt the Zymolyase reaction. Tetrads were dissected on YPD plates using a Singer SporePlay+ microscope (Singer Instruments). While many sporulated cultures had a mix of monads, dyads, and tetrads, only complete tetrads were dissected. *S. cerevisiae* spores were grown for 2–4 days at 30 °C, while *S. uvarum* spores were grown for an analogous time at room temperature. Spore viability was calculated as the number of spores that successfully returned to a vegetative state divided by the total number of spores dissected. A minimum number of 21 meioses (84 spores) were analyzed per temperature per strain; however, all but one sample successfully yielded 24 meioses (96 spores).

### Data analysis and visualization

Data analyses and visualizations were conducted using R (ver. 4.3.3) and RStudio (ver. 2024.04.0). Growth rates were analyzed using the R package “growthcurver (ver. 0.3.1)” (Sprouffske and Wagner 2016). Statistical analyses were done using R packages “dplyr (ver. 1.1.4),” “stats (ver. 4.3.3),” “FSA,” “car,” “multcomp,” and “rstatix (ver. 0.7.2),”(Wickham et al. 2023; R Core Team 2024; Ogle et al., (2025); Fox, Weisberg (2019); Hothorn et al. 2008; Kassambara (2023a)). Visualizations were constructed using the R packages “tidyverse (ver. 2.0.0),” “ggplot2 (ver. 3.5.1),” “viridis (ver. 0.6.5),” “wesanderson (ver. 0.3.7)”, “ggpubr (ver. 0.6.0)”,”ggsignif (ver. 0.6.4)”, and “ggtext (ver. 0.1.2)” (Wickham et al. 2019; Wickham 2016; Garnier et al. 2024; Ram, Wickham (2023); Kassambara (2023b); Constantin, Patil (2021); Wilke and Wiernik 2022). Flow cytometry gating and analysis were completed using FlowJo (ver. 10.0.0) (BD BioSciences).

## Data and resource availability

*S. cerevisiae* fluorescent tester strains are courtesy of Matthieu Falque, *S. uvarum* strains are courtesy of the Portuguese Yeast Culture Collection (PYCC) and Chris Hittinger. *S. uvarum* fluorescent tester strains are available from our lab upon request. Raw data generated and analyzed in this paper are included in the supplementary tables.

## Supplementary information

Supplementary Figures

Supplementary Tables

## References

[CR1] Aggarwal DD, Rybnikov S, Cohen I, Frenkel Z, Rashkovetsky E, Michalak P, Korol AB (2019) Desiccation-induced changes in recombination rate and crossover interference in Drosophila melanogaster: evidence for fitness-dependent plasticity. Genetica 147(3):291–302. 10.1007/s10709-019-00070-6. 31240599 10.1007/s10709-019-00070-6

[CR2] Almeida P, Gonçalves C, Teixeira S, Libkind D, Bontrager M, Masneuf-Pomarède I, Albertin W, Durrens P, Sherman DJ, Marullo P, Todd Hittinger C, Gonçalves P, Sampaio JP (2014) A Gondwanan imprint on global diversity and domestication of wine and cider yeast *Saccharomyces uvarum*. Nat Commun 5: 4044. 10.1038/ncomms5044. 24887054 10.1038/ncomms5044 PMC5081218

[CR3] AlZaben F, Chuong JN, Abrams MB, Brem RB (2021) Joint effects of genes underlying a temperature specialization tradeoff in yeast. PLOS Genet 17(9):e1009793. 10.1371/journal.pgen.1009793. 34520469 10.1371/journal.pgen.1009793 PMC8462698

[CR4] Baker EP, Peris D, Moriarty RV, Li XC, Fay JC, Hittinger CT (2019) Mitochondrial DNA and temperature tolerance in lager yeasts. Sci Adv 5(1):eaav1869. 10.1126/sciadv.aav1869. 30729163 10.1126/sciadv.aav1869 PMC6353617

[CR5] Bayliss MW, Riley R (1972) An analysis of temperature-dependent asynapsis in Triticum aestivum. Genet Res 20(2):193–200. 10.1017/S0016672300013707.

[CR6] Bilgir C, Dombecki CR, Chen PF, Villeneuve AM, Nabeshima K (2013) Assembly of the synaptonemal complex is a highly temperature-sensitive process that is supported by PGL-1 during Caenorhabditis elegans Meiosis. G3: Genes|Genomes|Genet 3(4):585–595. 10.1534/g3.112.005165. 10.1534/g3.112.005165 PMC3618346 23550120

[CR7] Bomblies K, Higgins JD, Yant L (2015) Meiosis evolves: Adaptation to external and internal environments. N Phytol 208(2):306–323. 10.1111/nph.13499. 10.1111/nph.13499 26075313

[CR8] Bursell M, Rohilla M, Ramirez L, Cheng Y, Schwarzkopf EJ, Guerrero RF, Heil CS (2024) Mixed outcomes in recombination rates after domestication: Revisiting theory and data (p. 2024.08.07.607048). bioRxiv. 10.1101/2024.08.07.607048 10.1111/mec.17773 PMC12353367 40271548

[CR9] Byers B, Goetsch L (1982) Reversible pachytene arrest of Saccharomyces cerevisiae at elevated temperature. Mol Gen Genet MGG 187(1):47–53. 10.1007/BF00384382. 6761544 10.1007/BF00384382

[CR10] Constantin A, Patil I (2021). “ggsignif: R package for displaying significance brackets for 'ggplot2'.” PsyArxiv. 10.31234/osf.io/7awm6, https://psyarxiv.com/7awm6.

[CR11] Cotton VE, Hoffmann ER, Abdullah MFF, Borts RH (2009) Interaction of Genetic and Environmental Factors in Saccharomyces cerevisiae Meiosis: The Devil is in the Details. In S. Keeney (Ed.), Meiosis: Volume 1, Molecular and Genetic Methods (pp. 3–20). Humana Press. 10.1007/978-1-59745-527-5_1 10.1007/978-1-59745-527-5_1 19799172

[CR12] Dapper AL, Payseur BA (2019) Molecular evolution of the meiotic recombination pathway in mammals. Evolution 73(12):2368–2389. 10.1111/evo.13850. 31579931 10.1111/evo.13850 PMC7050330

[CR13] Davidow LS, Byers B (1984) Enhanced gene conversion and postmeiotic segregation in pachytene-arrested Saccharomyces cerevisiae. Genetics 106(2):165–183. 10.1093/genetics/106.2.165. 6365687 10.1093/genetics/106.2.165 PMC1202250

[CR14] De Chiara M, Barré BP, Persson K, Irizar A, Vischioni C, Khaiwal S, Stenberg S, Amadi OC, Žun G, Doberšek K, Taccioli C, Schacherer J, Petrovič U, Warringer J, Liti G (2022) Domestication reprogrammed the budding yeast life cycle. Nat Ecol Evolut 6(4):Article 4. 10.1038/s41559-022-01671-9. 10.1038/s41559-022-01671-9 35210580

[CR15] Duan S-F, Han P-J, Wang Q-M, Liu W-Q, Shi J-Y, Li K, Zhang X-L, Bai F-Y (2018) The origin and adaptive evolution of domesticated populations of yeast from Far East Asia. Nat Commun 9(1):Article 1. 10.1038/s41467-018-05106-7. 10.1038/s41467-018-05106-7 PMC6043522 30002370

[CR16] Dumont BL (2020) Evolution: is recombination rate variation adaptive?. Curr Biol 30(8):R351–R353. 10.1016/j.cub.2020.02.061. 32315634 10.1016/j.cub.2020.02.061

[CR17] Dunham MJ, Gartenberg M, Brown GW (2015) Methods in Yeast Genetics and Genomics, 2015 Edition: A CSHL Course Manual. Cold Spring Harbor Laboratory Press.

[CR18] Elliott CG (1955) The effect of temperature on chiasma frequency. Heredity 9(3):Article 3. 10.1038/hdy.1955.39.

[CR19] Elrod SL, Chen SM, Schwartz K, Shuster EO (2009) Optimizing sporulation conditions for different Saccharomyces cerevisiae strain backgrounds. In S. Keeney (Ed.), Meiosis: Volume 1, Molecular and Genetic Methods (pp. 21–26). Humana Press. 10.1007/978-1-59745-527-5_2 10.1007/978-1-59745-527-5_2 19799173

[CR20] Esposito MS, Esposito RE (1969) The genetic control of sporulation in Saccharomyces I. The isolation of temperature-sensitive sporulation-deficient mutants. Genetics 61(1):79–89. 5802566 10.1093/genetics/61.1.79 PMC1212153

[CR21] Fan Q, Xu F, Petes TD (1995) Meiosis-specific double-strand DNA breaks at the HIS4 recombination hot spot in the yeast Saccharomyces cerevisiae: Control in cis and trans. Mol Cell Biol 15(3):1679–1688. 7862159 10.1128/mcb.15.3.1679 PMC230392

[CR22] Fox J, Weisberg S (2019) An R companion to applied regression, third edition. Sage, Thousand Oaks CA. https://www.john-fox.ca/Companion/.

[CR23] Gallone B, Steensels J, Prahl T, Soriaga L, Saels V, Herrera-Malaver B, Merlevede A, Roncoroni M, Voordeckers K, Miraglia L, Teiling C, Steffy B, Taylor M, Schwartz A, Richardson T, White C, Baele G, Maere S, Verstrepen KJ (2016) Domestication and Divergence of Saccharomyces cerevisiae Beer Yeasts. Cell 166(6):1397–1410.e16. 10.1016/j.cell.2016.08.020. 27610566 10.1016/j.cell.2016.08.020 PMC5018251

[CR24] Garnier S, Ross N, Rudis R, Camargo PA, Sciaini M, Scherer C (2024) viridis(Lite)—Colorblind-friendly color maps for R. 10.5281/zenodo.4679423, viridis package version 0.6.5, https://sjmgarnier.github.io/viridis/.

[CR25] Gerke JP, Chen CTL, Cohen BA (2006) Natural isolates of saccharomyces cerevisiae display complex genetic variation in sporulation efficiency. Genetics 174(2):985–997. 10.1534/genetics.106.058453. 16951083 10.1534/genetics.106.058453 PMC1602093

[CR26] Gonçalves P, Valério E, Correia C, Almeida JMGCFde, Sampaio JP (2011) Evidence for divergent evolution of growth temperature preference in sympatric Saccharomyces species. PLoS One 6(6):e20739. 10.1371/journal.pone.0020739. 21674061 10.1371/journal.pone.0020739 PMC3107239

[CR27] Henderson IR, Bomblies K (2021) Evolution and plasticity of genome-wide meiotic recombination rates. Annu Rev Genet 55:23–43. 10.1146/annurev-genet-021721-033821. 34310193 10.1146/annurev-genet-021721-033821

[CR28] Higgins JD, Perry RM, Barakate A, Ramsay L, Waugh R, Halpin C, Armstrong SJ, Franklin FCH (2012) Spatiotemporal asymmetry of the meiotic program underlies the predominantly distal distribution of meiotic crossovers in Barley. Plant Cell 24(10):4096–4109. 10.1105/tpc.112.102483. 23104831 10.1105/tpc.112.102483 PMC3517238

[CR29] Hothorn T, Bretz F, Westfall P (2008) Simultaneous inference in general parametric models. Biometrical J 50(3):346–363. 10.1002/bimj.200810425 18481363

[CR30] Iossa G (2019) Sex-specific differences in thermal fertility limits. Trends Ecol Evolut 34(6):490–492. 10.1016/j.tree.2019.02.016. 10.1016/j.tree.2019.02.016 30910426

[CR31] Jambhekar A, Amon A (2008) Control of meiosis by respiration. Curr Biol 18(13):969–975. 10.1016/j.cub.2008.05.047. 18595705 10.1016/j.cub.2008.05.047 PMC2504020

[CR32] Johnston JR, Mortimer R (1967) Influence of temperature on recombination in yeast. Heredity 22(2):297–303. 10.1038/hdy.1967.33. 5235105 10.1038/hdy.1967.33

[CR33] Kassambara A (2023a) ggpubr: 'ggplot2' Based Publication Ready Plots. R package version 0.6.0, https://rpkgs.datanovia.com/ggpubr/.

[CR34] Kassambara A (2023b) rstatix: Pipe-Friendly Framework for Basic Statistical Tests. R package version 0.7.2, https://rpkgs.datanovia.com/rstatix/.

[CR35] Kohl KP, Singh ND (2018) Experimental evolution across different thermal regimes yields genetic divergence in recombination fraction but no divergence in temperature associated plastic recombination. Evolution 72(4):989–999. 10.1111/evo.13454. 29468654 10.1111/evo.13454

[CR36] Korol AB, Iliadi KG (1994) Increased recombination frequencies resulting from directional selection for geotaxis in Drosophila. Heredity 72(Pt 1):64–68. 10.1038/hdy.1994.7. 8119830 10.1038/hdy.1994.7

[CR37] Langdon QK, Peris D, Eizaguirre JI, Opulente DA, Buh KV, Sylvester K, Jarzyna M, Rodríguez ME, Lopes CA, Libkind D, Hittinger CT (2020) Postglacial migration shaped the genomic diversity and global distribution of the wild ancestor of lager-brewing hybrids. PLOS Genet 16(4):e1008680. 10.1371/journal.pgen.1008680. 32251477 10.1371/journal.pgen.1008680 PMC7162524

[CR38] Leducq J-B, Charron G, Samani P, Dubé AK, Sylvester K, James B, Almeida P, Sampaio JP, Hittinger CT, Bell G, Landry CR (2014) Local climatic adaptation in a widespread microorganism. Proc R Soc B: Biol Sci 281(1777):20132472. 10.1098/rspb.2013.2472. 10.1098/rspb.2013.2472 PMC3896012 24403328

[CR39] Lee TJ, Liu Y, Liu W-A, Lin Y-F, Lee H-H, Ke H-M, Huang J-P, Lu M-YJ, Hsieh C-L, Chung K-F, Liti G, Tsai IJ (2022) Extensive sampling of Saccharomyces cerevisiae in Taiwan reveals ecology and evolution of predomesticated lineages. Genome Res. gr.276286.121. 10.1101/gr.276286.121 10.1101/gr.276286.121 PMC9104698 35361625

[CR40] Lenormand T, Engelstädter J, Johnston SE, Wijnker E, Haag CR (2016) Evolutionary mysteries in meiosis. Philos Trans R Soc B: Biol Sci 371(1706):20160001. 10.1098/rstb.2016.0001. 10.1098/rstb.2016.0001 PMC5031626 27619705

[CR41] Li XC, Peris D, Hittinger CT, Sia EA, Fay JC (2019) Mitochondria-encoded genes contribute to evolution of heat and cold tolerance in yeast. Sci Adv 5(1):eaav1848. 10.1126/sciadv.aav1848. 30729162 10.1126/sciadv.aav1848 PMC6353624

[CR42] Lim JGY, Stine RRW, Yanowitz JL (2008) Domain-specific regulation of recombination in Caenorhabditis elegans in response to temperature, age and sex. Genetics 180(2):715–726. 10.1534/genetics.108.090142. 18780748 10.1534/genetics.108.090142 PMC2567375

[CR43] Liti G, Carter DM, Moses AM, Warringer J, Parts L, James SA, Davey RP, Roberts IN, Burt A, Koufopanou V, Tsai IJ, Bergman CM, Bensasson D, O’Kelly MJT, van Oudenaarden A, Barton DBH, Bailes E, Nguyen AN, Jones M, Louis EJ (2009) Population genomics of domestic and wild yeasts. Nature 458(7236):337–341. 10.1038/nature07743. 19212322 10.1038/nature07743 PMC2659681

[CR44] Lloyd A, Morgan C, H Franklin FC, Bomblies K (2018) Plasticity of meiotic recombination rates in response to temperature in arabidopsis. Genetics 208(4):1409–1420. 10.1534/genetics.117.300588. 29496746 10.1534/genetics.117.300588 PMC5887139

[CR45] Loidl J (1989) Effects of elevated temperature on meiotic chromosome synapsis in Allium ursinum. Chromosoma 97(6):449–458. 10.1007/BF00295029.

[CR96] Lowry DB, Rockwood RC, Willis JH (2008). Ecological Reproductive Isolation of Coast and Inland Races of Mimulus Guttatus. Evolution 62:2196–2214. 10.1111/j.1558-5646.2008.00457.x PMC11110535 18637837

[CR97] Macnair M, Gardner M (1998). Chapter 12. The Evolution of Edaphic Endemics. In: Howard DJ, Berlocher SH (eds) Endless Forms. Species and Speciation, Oxford University Press, New York, pp 157–171.

[CR46] Modliszewski JL, Wang H, Albright AR, Lewis SM, Bennett AR, Huang J, Ma H, Wang Y, Copenhaver GP (2018) Elevated temperature increases meiotic crossover frequency via the interfering (Type I) pathway in Arabidopsis thaliana. PLOS Genet 14(5):e1007384. 10.1371/journal.pgen.1007384. 29771908 10.1371/journal.pgen.1007384 PMC5976207

[CR47] Molinet J, Stelkens R (2025) The evolution of thermal performance curves in response to rising temperatures across the model genus yeast. Proc Natl Acad Sci 122(21):e2423262122. 10.1073/pnas.2423262122. 40392856 10.1073/pnas.2423262122 PMC12130858

[CR48] Morgan C, Zhang H, Bomblies K (2017) Are the effects of elevated temperature on meiotic recombination and thermotolerance linked via the axis and synaptonemal complex?. Philos Trans R Soc B: Biol Sci 372(1736):20160470. 10.1098/rstb.2016.0470. 10.1098/rstb.2016.0470 PMC5698628 29109229

[CR49] Murphy HA, Zeyl CW (2012) Prezygotic isolation between *Saccharomyces cerevisiae* and *Saccharomyces paradoxus* through differences in mating speed and germination timing. Evolution 66(4):1196–1209. 10.1111/j.1558-5646.2011.01516.x. 22486698 10.1111/j.1558-5646.2011.01516.x

[CR50] Murphy HA, Kuehne HA, Francis CA, Sniegowski PD (2006) Mate choice assays and mating propensity differences in natural yeast populations. Biol Lett 2(4):553–556. 10.1098/rsbl.2006.0534. 17148286 10.1098/rsbl.2006.0534 PMC1833990

[CR51] Naumov GI, James SA, Naumova ES, Louis EJ, Roberts IN (2000) Three new species in the Saccharomyces sensu stricto complex: Saccharomyces cariocanus, Saccharomyces kudriavzevii and Saccharomyces mikatae. Int J Syst Evol Microbiol 50(Pt 5):1931–1942. 10.1099/00207713-50-5-1931. 11034507 10.1099/00207713-50-5-1931

[CR52] Nebel BR, Hackett EM (1961) Synaptinemal complexes (Cores) in primary spermatocytes of mouse under elevated temperature. Nature 190(4774):Article 4774. 10.1038/190467a0. 10.1038/190467a0 13728068

[CR53] Ogle DH, Doll JC, Wheeler AP, Dinno A (2025) FSA: simple fisheries stock assessment methods. R package version 0.9.6, https://CRAN.R-project.org/package=FSA.

[CR54] Ono J, Greig D, Boynton PJ (2020) Defining and disrupting species boundaries in Saccharomyces. Annu Rev Microbiol 74:477–495. 10.1146/annurev-micro-021320-014036. 32689915 10.1146/annurev-micro-021320-014036

[CR55] Pao WK, Li HW (1948) Desynapsis and other abnormalities induced by high temperature. J Genet 48(3):297–310. 10.1007/BF02986629. 18905078 10.1007/BF02986629

[CR56] Peris D, Alexander WG, Fisher KJ et al. (2020) Synthetic hybrids of six yeast species. Nat Commun 11: 2085. 10.1038/s41467-020-15559-4. 32350251 10.1038/s41467-020-15559-4 PMC7190663

[CR57] Peris D, Ubbelohde EJ, Kuang MC, Kominek J, Langdon QK, Adams M, Koshalek JA, Hulfachor AB, Opulente DA, Hall DJ, Hyma K, Fay JC, Leducq J-B, Charron G, Landry CR, Libkind D, Gonçalves C, Gonçalves P, Sampaio JP, Hittinger CT (2022) Macroevolutionary diversity of traits and genomes in the model yeast genus Saccharomyces (p 2022.03.30.486421). bioRxiv. 10.1101/2022.03.30.486421

[CR58] Peter J, De Chiara M, Friedrich A, Yue J-X, Pflieger D, Bergström A, Sigwalt A, Barre B, Freel K, Llored A, Cruaud C, Labadie K, Aury J-M, Istace B, Lebrigand K, Barbry P, Engelen S, Lemainque A, Wincker P, Schacherer J (2018) Genome evolution across 1,011 Saccharomyces cerevisiae isolates. Nature 556(7701):339–344. 10.1038/s41586-018-0030-5. 29643504 10.1038/s41586-018-0030-5 PMC6784862

[CR59] Plough HH (1917) The effect of temperature on crossingover in Drosophila. J Exp Zool 24(2):147–209. 10.1002/jez.1400240202.

[CR60] R Core Team (2024) _R: A Language and Environment for Statistical Computing_. R Foundation for Statistical Computing, Vienna, Austria. https://www.R-project.org/.

[CR61] Raffoux X, Bourge M, Dumas F, Martin OC, Falque M (2018a) High-throughput measurement of recombination rates and genetic interference in Saccharomyces cerevisiae. Yeast 35(6):431–442. 10.1002/yea.3315. 29577404 10.1002/yea.3315

[CR62] Raffoux X, Bourge M, Dumas F, Martin OC, Falque M (2018b) Role of Cis, Trans, and Inbreeding Effects on Meiotic Recombination in Saccharomyces cerevisiae. Genetics 210(4):1213–1226. 10.1534/genetics.118.301644. 30291109 10.1534/genetics.118.301644 PMC6283170

[CR63] Ram K, Wickham H (2023) Wesanderson: A Wes Anderson Palette Generator. R package version 0.3.7, https://github.com/karthik/wesanderson.

[CR64] Robinson HA, Pinharanda A, Bensasson D (2016) Summer temperature can predict the distribution of wild yeast populations. Ecol Evolut 6(4):1236–1250. 10.1002/ece3.1919. 10.1002/ece3.1919 PMC4761769 26941949

[CR65] Rogers DW, Denton JA, McConnell E, Greig D (2015) Experimental evolution of species recognition. Curr Biol: CB 25(13):1753–1758. 10.1016/j.cub.2015.05.023. 26073134 10.1016/j.cub.2015.05.023

[CR66] Rogers DW, McConnell E, Ono J, Greig D (2018) Spore-autonomous fluorescent protein expression identifies meiotic chromosome mis-segregation as the principal cause of hybrid sterility in yeast. PLOS Biol 16(11):e2005066. 10.1371/journal.pbio.2005066. 30419022 10.1371/journal.pbio.2005066 PMC6258379

[CR67] Rose AM, Baillie DL (1979) The effect of temperature and parental age on recombination and nondisjunction in Caenorhabditis elegans. Genetics 92(2):409–418. 10.1093/genetics/92.2.409. 17248928 10.1093/genetics/92.2.409 PMC1213967

[CR68] Rybnikov SR, Frenkel ZM, Korol AB (2017) What drives the evolution of condition-dependent recombination in diploids? Some insights from simulation modelling. Philos Trans R Soc B Biol Sci 372(1736):20160460. 10.1098/rstb.2016.0460. 10.1098/rstb.2016.0460 PMC5698622 29109223

[CR69] Salvadó Z, Arroyo-López FN, Guillamón JM, Salazar G, Querol A, Barrio E (2011) Temperature adaptation markedly determines evolution within the genus Saccharomyces. Appl Environ Microbiol 77(7):2292–2302. 10.1128/AEM.01861-10. 21317255 10.1128/AEM.01861-10 PMC3067424

[CR70] Sampaio JP, Gonçalves P (2008) Natural populations of Saccharomyces kudriavzevii in Portugal Are Associated with Oak Bark and Are Sympatric with S. cerevisiae and S. paradoxus. Appl Environ Microbiol 74(7):2144–2152. 10.1128/AEM.02396-07. 18281431 10.1128/AEM.02396-07 PMC2292605

[CR71] Samuk K, Manzano-Winkler B, Ritz KR, Noor MAF (2020) Natural Selection Shapes Variation in Genome-wide Recombination Rate in Drosophila pseudoobscura Curr Biol 30:1517–1528. 32275873 10.1016/j.cub.2020.03.053

[CR72] Schwarzkopf EJ, Brandt N, Heil CS (2024) The recombination landscape of introgression in yeast (p. 2024.01.04.574263). bioRxiv. 10.1101/2024.01.04.574263 10.1371/journal.pgen.1011585 PMC11845044 39937775

[CR73] Smith HF (1936) Influence of temperature on crossing-over in Drosophila. Nature 138(3486):329–330. 10.1038/138329b0.

[CR74] Sprouffske K, Wagner A (2016) Growthcurver: an R package for obtaining interpretable metrics from microbial growth curves. BMC Bioinforma 17(1):172. 10.1186/s12859-016-1016-7. 10.1186/s12859-016-1016-7 PMC4837600 27094401

[CR75] Stevison LS, Sefick S, Rushton C, Graze RM (2017) Recombination rate plasticity: revealing mechanisms by design. Philos Trans R Soc B Biol Sci 372(1736):20160459. 10.1098/rstb.2016.0459. 10.1098/rstb.2016.0459 PMC5698621 29109222

[CR76] Sweeney JY, Kuehne HA, Sniegowski PD (2004) Sympatric natural Saccharomyces cerevisiae and S. paradoxus populations have different thermal growth profiles. FEMS Yeast Res 4(4–5):521–525. 10.1016/S1567-1356(03)00171-5. 14734033 10.1016/S1567-1356(03)00171-5

[CR77] Tomar P, Bhatia A, Ramdas S, Diao L, Bhanot G, Sinha H (2013) Sporulation genes associated with sporulation efficiency in natural isolates of yeast. PLOS One 8(7):e69765. 10.1371/journal.pone.0069765. 23874994 10.1371/journal.pone.0069765 PMC3714247

[CR78] Trainor BM, Ciccaglione K, Czymek M, Law MJ (2021) Distinct requirements for the COMPASS core subunits Set1, Swd1, and Swd3 during meiosis in the budding yeast Saccharomyces cerevisiae. G3 Genes Genomes Genet 11(11):jkab283. 10.1093/g3journal/jkab283. 10.1093/g3journal/jkab283 PMC8527496 34849786

[CR79] Turner TL, Levine MT, Eckert ML, Begun DJ (2008) Genomic analysis of adaptive differentiation in Drosophila melanogaster. Genetics 179(1):455–473. 10.1534/genetics.107.083659. 18493064 10.1534/genetics.107.083659 PMC2390623

[CR80] van Heerwaarden B, Sgrò CM (2021) Male fertility thermal limits predict vulnerability to climate warming. Nat Commun 12(1):Article 1. 10.1038/s41467-021-22546-w. 10.1038/s41467-021-22546-w PMC8044094 33850157

[CR98] Vasek FC, Sauer RH (1971) Seasonal Progression of Flowering in Clarkia. Ecology 52:1038–1045.

[CR81] Walsh BS, Parratt SR, Hoffmann AA, Atkinson D, Snook RR, Bretman A, Price TAR (2019) The impact of climate change on fertility. Trends Ecol Evolut 34(3):249–259. 10.1016/j.tree.2018.12.002. 10.1016/j.tree.2018.12.002 30635138

[CR82] Weiss CV, Roop JI, Hackley RK, Chuong JN, Grigoriev IV, Arkin AP, Skerker JM, Brem RB (2018) Genetic dissection of interspecific differences in yeast thermotolerance. Nat Genet 50(11):1501–1504. 10.1038/s41588-018-0243-4. 30297967 10.1038/s41588-018-0243-4 PMC6430122

[CR83] Weitz AP, Dukic M, Zeitler L, Bomblies K (2021) Male meiotic recombination rate varies with seasonal temperature fluctuations in wild populations of autotetraploid Arabidopsis arenosa. Mol Ecol 30(19):4630–4641. 10.1111/mec.16084. 34273213 10.1111/mec.16084 PMC9292783

[CR84] Wickham H (2016) ggplot2: Elegant Graphics for Data Analysis. Springer-Verlag New York. ISBN 978-3-319-24277-4, https://ggplot2.tidyverse.org.

[CR85] Wickham H, Averick M, Bryan J, Chang W, McGowan LD, François R, Grolemund G, Hayes A, Henry L, Hester J, Kuhn M, Pedersen TL, Miller E, Bache SM, Müller K, Ooms J, Robinson D, Seidel DP, Spinu V, Takahashi K, Vaughan D, Wilke C, Woo K, Yutani H (2019) Welcome to the tidyverse. J Open Source Softw 4(43):1686. 10.21105/joss.01686.

[CR86] Wickham H, François R, Henry L, Müller K, Vaughan D (2023) dplyr: a grammar of data manipulation. R package version 1.1.4, https://github.com/tidyverse/dplyr, https://dplyr.tidyverse.org.

[CR87] Wilke C, Wiernik B (2022) ggtext: improved text rendering support for 'ggplot2'. R package version 0.1.2, https://github.com/wilkelab/ggtext.

[CR88] Wilkins AS, Holliday R (2009) The evolution of meiosis from mitosis. Genetics 181(1):3–12. 10.1534/genetics.108.099762. 19139151 10.1534/genetics.108.099762 PMC2621177

[CR89] Winbush A, Singh ND (2021) Genomics of recombination rate variation in temperature-evolved Drosophila melanogaster Populations. Genome Biol Evolut 13(1):evaa252. 10.1093/gbe/evaa252. 10.1093/gbe/evaa252 PMC7851596 33247719

[CR90] Wooliver R, Vtipilthorpe EE, Wiegmann AM, Sheth SN (2022) A viewpoint on ecological and evolutionary study of plant thermal performance curves in a warming world. AoB PLANTS 14(3):plac016. 10.1093/aobpla/plac016. 35615255 10.1093/aobpla/plac016 PMC9126585

[CR91] Wright KM, Arnold B, Xue K, Šurinová M, O’Connell J, Bomblies K (2015) Selection on meiosis genes in diploid and tetraploid Arabidopsis arenosa. Mol Biol Evolut 32(4):944–955. 10.1093/molbev/msu398. 10.1093/molbev/msu398 PMC4379401 25543117

[CR92] Yazawa T, Nakayama Y, Fujimoto K, Matsuda Y, Abe K, Kitano T, Abé S-I, Yamamoto T (2003) Abnormal spermatogenesis at low temperatures in the Japanese red-bellied newt, Cynops pyrrhogaster: possible biological significance of the cessation of spermatocytogenesis. Mol Reprod Dev 66(1):60–66. 10.1002/mrd.10328. 12874800 10.1002/mrd.10328

[CR93] Zhang K, Wu X-C, Zheng D-Q, Petes TD (2017) Effects of Temperature on the Meiotic Recombination Landscape of the Yeast Saccharomyces cerevisiae. mBio 8(6):e02099-17. 10.1128/mBio.02099-17. 29259092 10.1128/mBio.02099-17 PMC5736917

[CR94] Zhao H, Wang Q, Liu C, Shang Y, Wen F, Wang F, Liu W, Xiao W, Li W (2018) A Role for the Respiratory Chain in Regulating Meiosis Initiation in Saccharomyces cerevisiae. Genetics 208(3):1181–1194. 10.1534/genetics.118.300689. 29301906 10.1534/genetics.118.300689 PMC5844330

[CR95] Zheng T, Nibau C, Phillips DW, Jenkins G, Armstrong SJ, Doonan JH (2014) CDKG1 protein kinase is essential for synapsis and male meiosis at high ambient temperature in Arabidopsis thaliana. Proc Natl Acad Sci 111(6):2182–2187. 10.1073/pnas.1318460111. 24469829 10.1073/pnas.1318460111 PMC3926089

