# Supplementary material for: Impacts of temperature on recombination rate and meiotic success in thermotolerant and cold-tolerant yeast species
Source: Heredity (Edinb). 2025 Jul 26;134(8):473–84. doi: 10.1038/s41437-025-00778-6 (PMC12316985; doi:10.1038/s41437-025-00778-6)
Supplement: Supplementary file 1 — Supplementary Figures [file 41437_2025_778_MOESM1_ESM.docx]

**Figure S1**


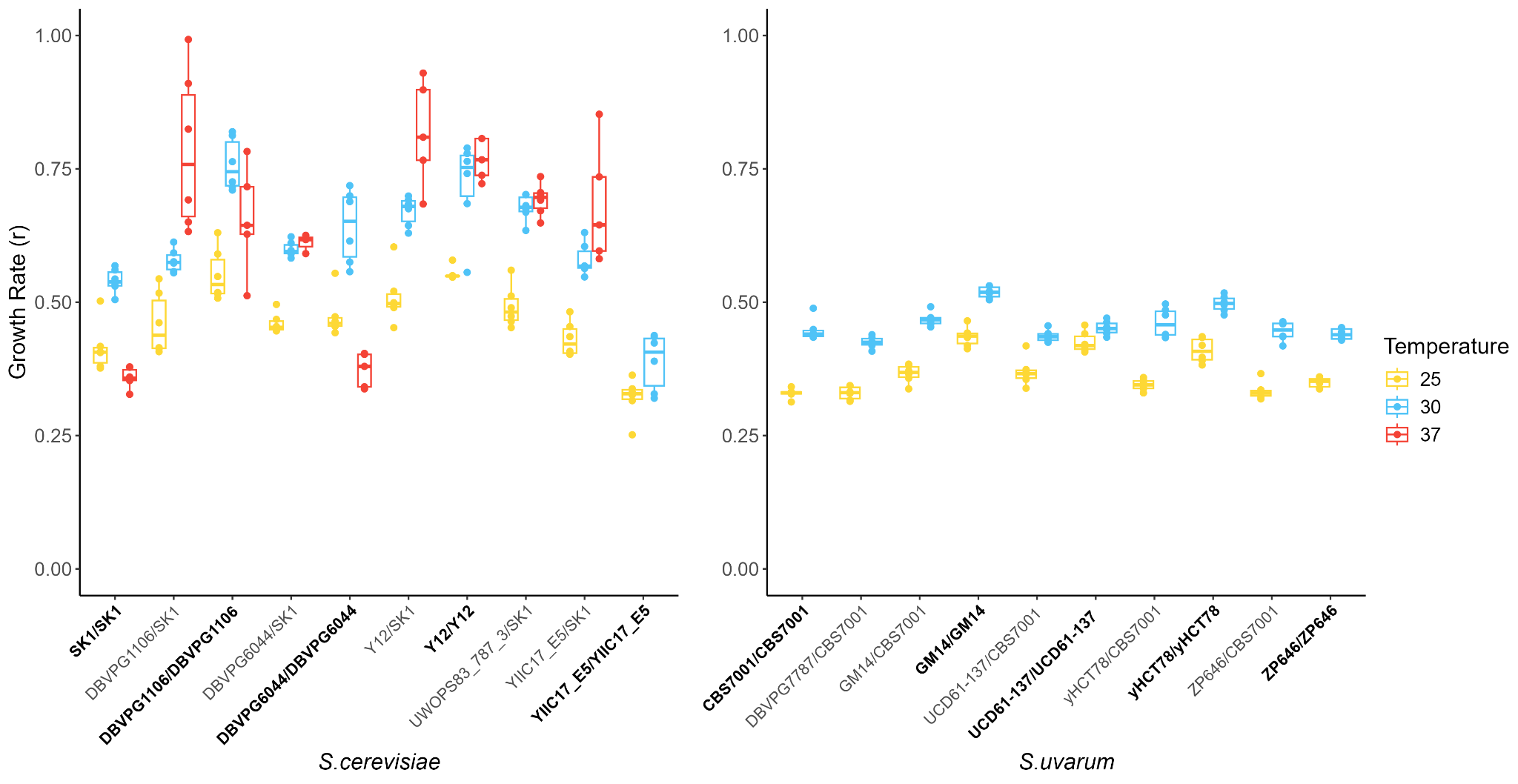
Growth rate (r) as calculated for the mitotic growth of each strain in liquid YPD. Bolded strains denote “pure” diploids where both parents are of the same strain background; unbolded strains represent crosses between a fluorescently marked haploid of a lab strain background and a haploid from a strain of interest. At 37°C, *S.cerevisiae* strain YIIC17_E5/YIIC17_E5 and *S.uvarum* strains failed to produce a measurable growth curve; thus, these data have been omitted from those plotted.

**Figure S2**

**
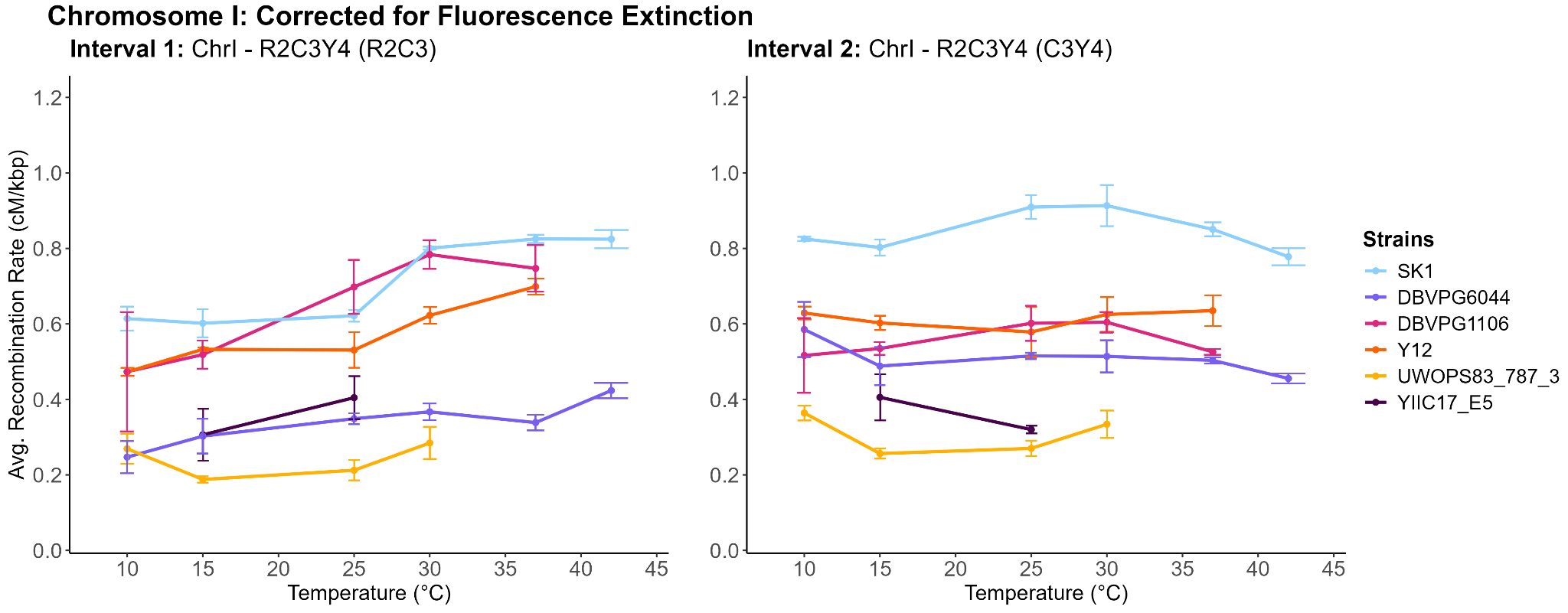
**

Average recombination rate (in cM/kbp) as calculated for intervals 1 and 2 from sporulated crosses with the ChrI-R2C3Y4 fluorescent tester strain at all viable temperatures. Strain name refers to the parent strain crossed with this fluorescent tester to produce a hybrid diploid. Recombination rate estimates were corrected for fluorescence extinction using a maximum likelihood model derived in Raffoux et al 2018a. Error bars indicate standard deviation above and below the mean, as calculated between biological replicates.

**Figure S3**

**
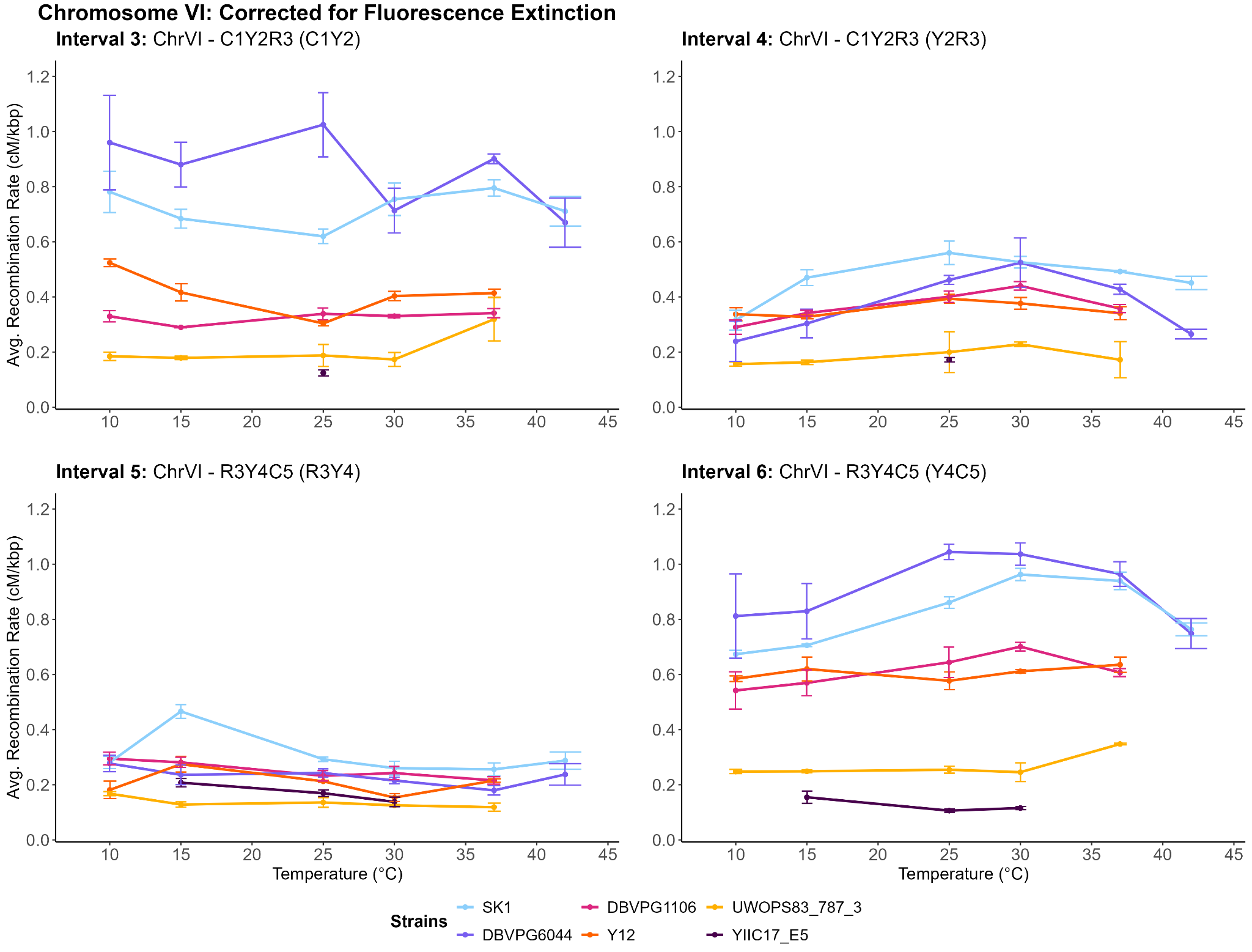
**

Average recombination rate (in cM/kbp) as calculated for intervals 3, 4 ,5 and 6 from sporulated crosses with the ChrVI-C1Y2R3 fluorescent tester strain (3 and 4) and the ChrVI-R3Y4C5 fluorescent tester strain (5 and 6) at all viable temperatures. Strain name refers to the parent strain crossed with each fluorescent tester to produce a hybrid diploid. Recombination rate estimates were corrected for fluorescence extinction using a maximum likelihood model derived in Raffoux et al 2018a. Error bars indicate standard deviation above and below the mean, as calculated between biological replicates.

**Figure S4**

**
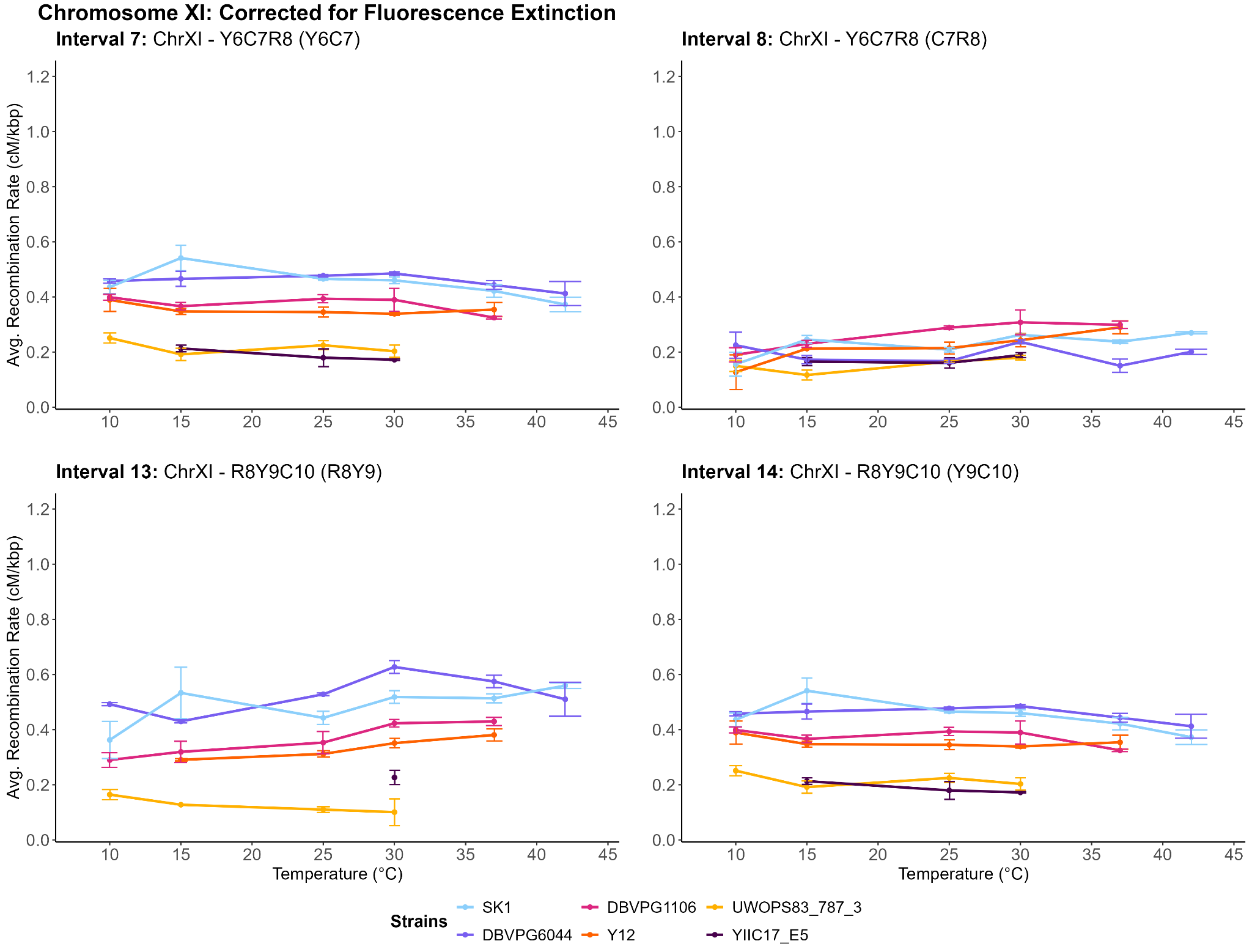
**

Average recombination rate (in cM/kbp) as calculated for intervals 7, 8 ,13 and 14 from sporulated crosses with the ChrXI-Y6C7R8 fluorescent tester strain (7 and 8) and the ChrXI-R8Y9C10 fluorescent tester strain (13 and 14) at all viable temperatures. Strain name refers to the parent strain crossed with each fluorescent tester to produce a hybrid diploid. Recombination rate estimates were corrected for fluorescence extinction using a maximum likelihood model derived in Raffoux et al 2018a. Error bars indicate standard deviation above and below the mean, as calculated between biological replicates.

**Figure S5**


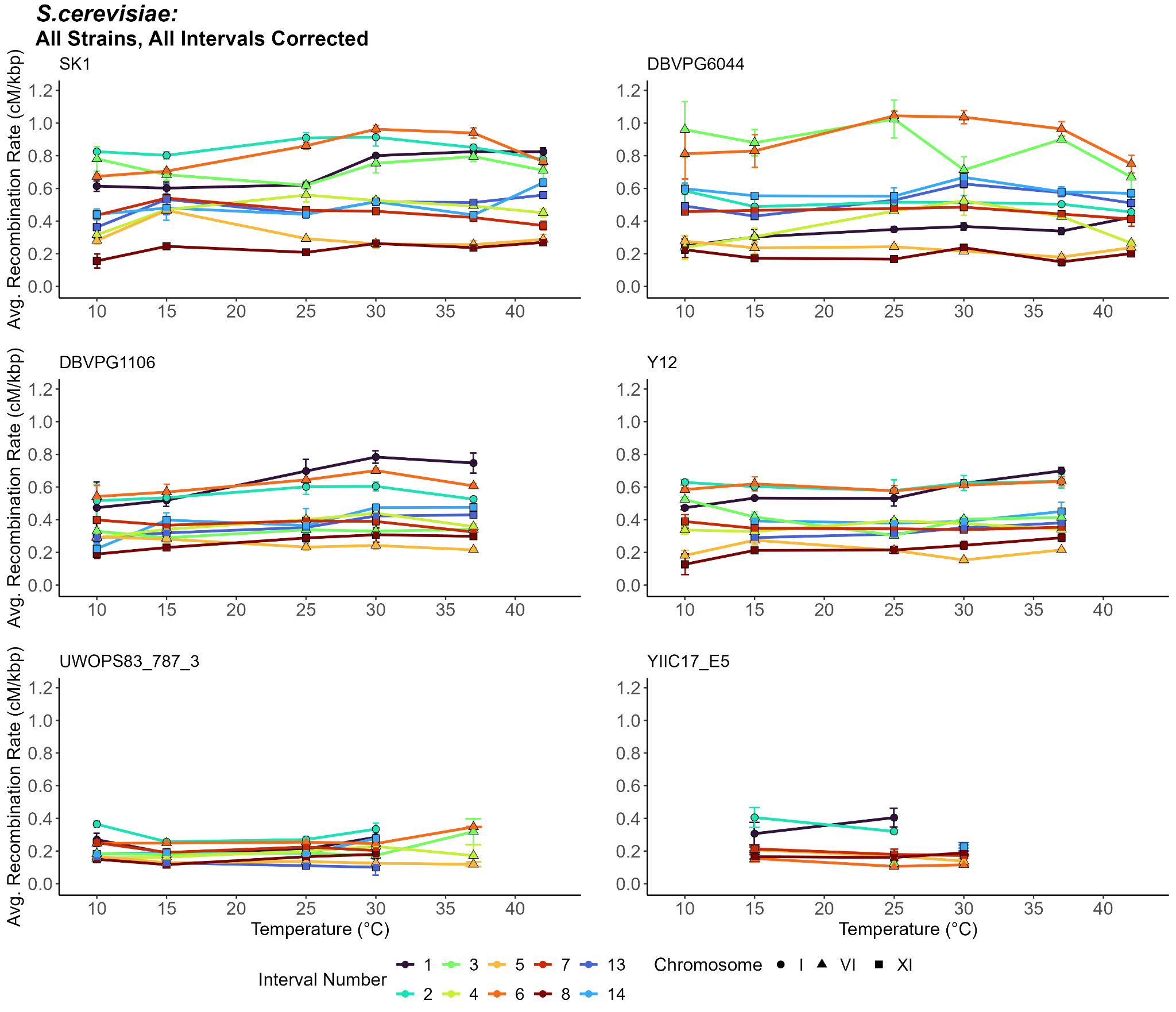


Average recombination rate (in cM/kbp) as calculated for all measured intervals at all viable temperatures, organized by strain. Strain name refers to the parent strain crossed with each fluorescent tester to produce a hybrid diploid. Recombination rate estimates were corrected for fluorescence extinction using a maximum likelihood model derived in Raffoux et al 2018a. Colors differentiate intervals, while shapes indicate the chromosome on which each interval is located. Error bars indicate standard deviation above and below the mean, as calculated between biological replicates.

**Figure S6**


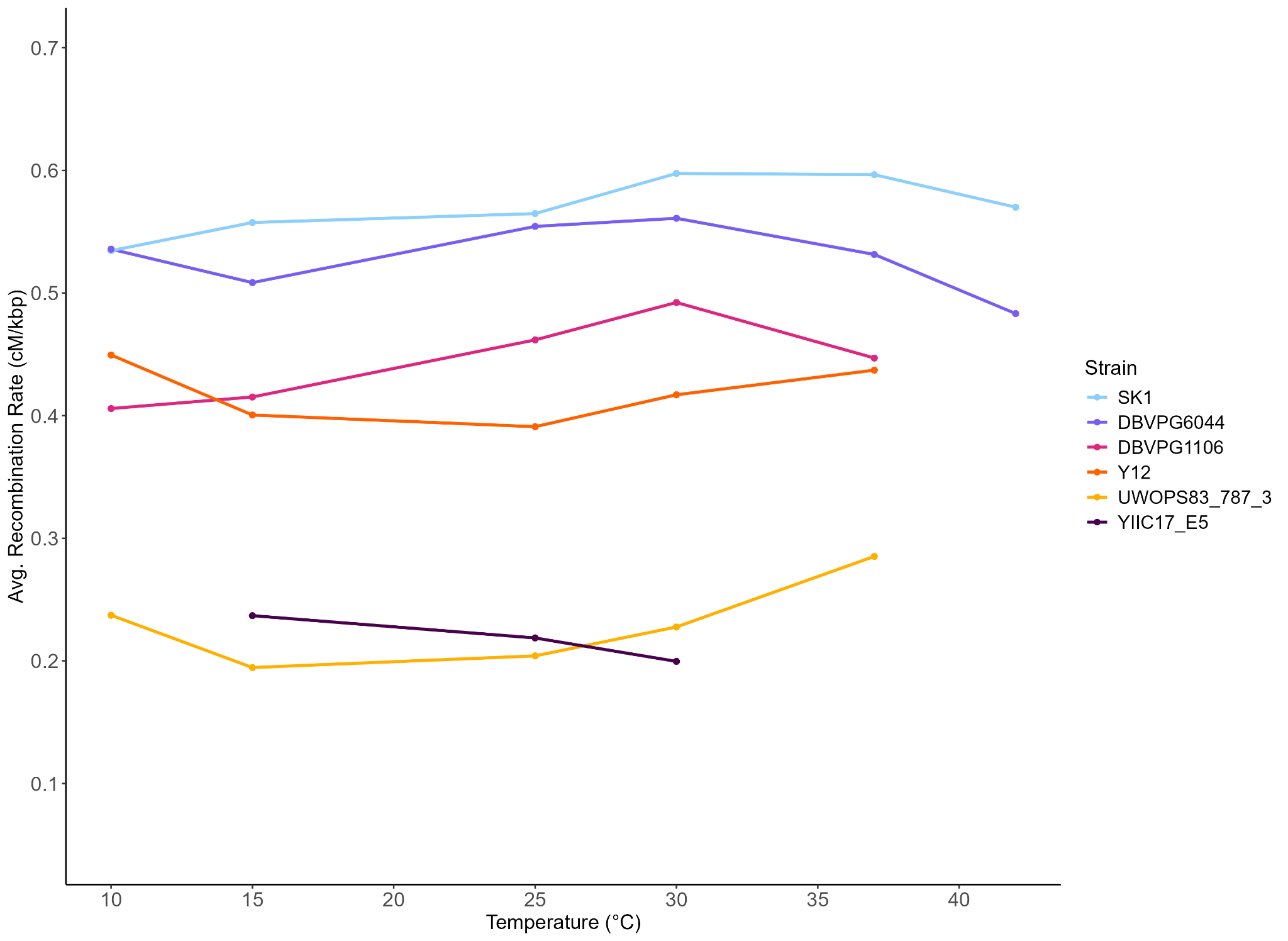


Average recombination rate (cM/kbp) as calculated from data pooled across all measured intervals for each *S. cerevisiae* strain at each temperature. In accordance with previous terminology utilized in Raffoux et al 2018b, we refer to this measure as the “global” recombination rate. For our study, this calculation includes a maximum of 10 intervals across chromosomes I, VI, and XI. Note, due to strain-specific complications, not every interval of these 10 was measured at each temperature and included in this calculation.
